# Regulation of TAR DNA binding protein 43 (TDP-43) homeostasis by cytosolic DNA accumulation

**DOI:** 10.1016/j.jbc.2024.107999

**Published:** 2024-11-15

**Authors:** Cha Yang, Cynthia Leifer, Jan Lammerding, Fenghua Hu

**Affiliations:** 1 Department of Molecular Biology and Genetics, Weill Institute for Cell and Molecular Biology, Cornell University, Ithaca, New York, USA; 2 Department of Microbiology and Immunology, Cornell University, Ithaca, New York, USA; 3 Department of Biomedical Engineering, Weill Institute for Cell and Molecular Biology, Ithaca, New York, USA

**Keywords:** TDP-43, DNA molecules, phase separation, proteinopathy, lamin

## Abstract

TAR DNA-binding protein 43 (TDP-43) is a DNA/RNA binding protein predominantly localized in the nucleus under physiological conditions. TDP-43 proteinopathy, characterized by cytoplasmic aggregation and nuclear loss, is associated with many neurodegenerative diseases, including amyotrophic lateral sclerosis (ALS) and frontotemporal lobar degeneration (FTLD). Thus it is crucial to understand the molecular mechanism regulating TDP-43 homeostasis. Here, we show that the uptake of oligodeoxynucleotides (ODNs) from the extracellular space induces reversible TDP-43 cytoplasmic puncta formation in both neurons and glia. ODNs facilitate the liquid-liquid phase separation of TDP-43 in *vitro*. Importantly, persistent accumulation of DNA in the cytoplasm leads to nuclear depletion of TDP-43 and enhanced production of a short isoform of TDP-43 (sTDP-43). In addition, in response to ODN uptake, the nuclear import receptor karyopherin subunit β1 (KPNB1) is sequestered in the cytosolic TDP-43 puncta. ALS-linked Q331K mutation decreases the dynamics of cytoplasmic TDP-43 puncta and increases the levels of sTDP-43. Moreover, the TDP-43 cytoplasmic puncta are induced by DNA damage and by impaired nuclear envelope integrity due to Lamin A/C deficiency. In summary, our data support that abnormal DNA accumulation in the cytoplasm may be one of the key mechanisms leading to TDP-43 proteinopathy and provides novel insights into molecular mechanisms of ALS caused by TDP-43 mutations.

TAR DNA-binding protein 43 (TDP-43), encoded by the *Tardbp* gene, is mostly found in the nucleus but shuttles between the nucleus and cytoplasm to regulate RNA metabolism, such as transcription, translation, pre-mRNA splicing, and mRNA stability (1, 2, 3, 4). TDP-43 proteinopathy characterized by the cytoplasmic aggregation and nuclear depletion of TDP-43 is a common pathological feature of several neurodegenerative diseases, including amyotrophic lateral sclerosis (ALS), frontotemporal lobar degeneration (FTLD), and Alzheimer’s disease (AD) (1, 4, 5). Thus, unraveling the molecular mechanisms leading to cytoplasmic TDP-43 aggregation is critical for understanding the mechanisms of neurodegenerative diseases associated with TDP-43 proteinopathy. However, the molecular mechanism leading to TDP-43 aggregation is still unclear.

TDP-43 is comprised of an N-terminal domain, two DNA/RNA recognition motifs (RRMs), and a C-terminal prion-like low complexity domain (PrLD) (4, 5). Under physiological conditions, TDP-43 is known to form liquid-like droplets through liquid-liquid phase separation (LLPS), mediated by the PrLD domain (6, 7). However, disease-related mutations, protein accumulation, or interaction with other proteins or nucleic acids affect the phase transition properties of TDP-43 and might trigger the formation of irreversible pathological aggregates seen in neurodegenerative diseases (8, 9, 10).

While the role of TDP-43 in RNA binding and RNA metabolism has been intensively studied, little is known about how its interaction with DNA molecules affects physiological functions and pathological aggregation of TDP-43. Here, we show that the accumulation of DNA in the cytosol drives the formation of cytoplasmic TDP-43 condensates. In addition, persistent DNA accumulation causes TDP-43 depletion from the nucleus and the production of a short TDP-43 isoform, suggesting a potential role of cytosolic DNA accumulation in TDP-43 pathology. ALS-linked Q331K mutation affects the dynamics of DNA-induced TDP-43 puncta. Additionally, cytoplasmic TDP-43 puncta can be induced by DNA damage or by compromised nuclear envelope integrity. Our studies uncover a previously unappreciated role of DNA binding in regulating TDP-43 homeostasis and provide novel insights into the molecular mechanisms of TDP-43 proteinopathy.

## Results

### Uptake of exogenous DNA molecules induces the formation of TDP-43 cytoplasmic puncta

**Figure 1:**
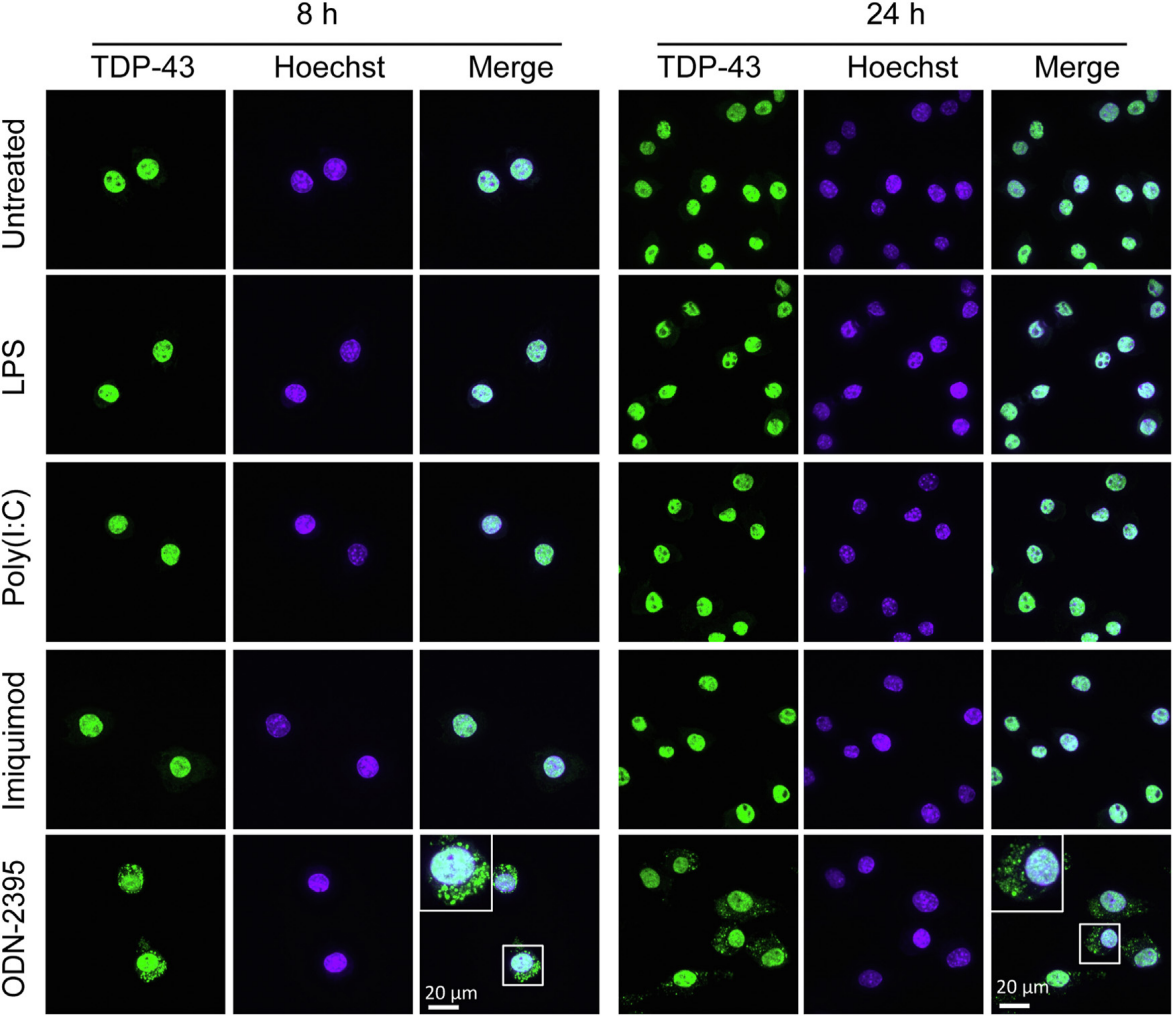
**CpG-ODN induces the TDP-43 cytoplasmic puncta formation.** RAW264.7 cells were untreated or treated with LPS, Poly (I: C), imiquimod, or CpG-ODN 2395 for 8 or 24 h, fixed, permeabilized with Triton-X100, and stained with mouse anti-TDP-43 antibodies. Representative images from three independent experiments were shown for each condition. Scale bar, 20 μm.

TDP-43 cytoplasmic aggregation and microglia-mediated inflammation are two pathologic hallmarks of many neurodegenerative diseases (11, 12, 13). To explore a potential connection between inflammation and cytoplasmic aggregation of TDP-43, we examined TDP-43 in the macrophage cell line RAW264.7 cells treated with different inflammatory stimuli, including lipopolysaccharide (LPS), polyinosinic-polycytidylic acid [(poly(I: C))], imiquimod, and oligodeoxynucleotide containing unmethylated CpG sequences (ODN 2395), ligands for Toll-like receptor (TLR) 4, 3, 7, and 9 (14), respectively. Among these, ODN-2395 triggered the formation of TDP-43 puncta in the cytoplasm after 8 and 24 h of treatment (Fig. 1). Because CpG-ODN is a ligand for TLR9 (15), we wondered whether TLR9 activation triggers TDP-43 cytoplasmic puncta formation. However, ablation of TLR9 did not prevent the formation of the cytoplasmic TDP-43 puncta upon CpG-ODN treatment (Fig. S1, *A* and *B*), indicating CpG-ODN induces the TDP-43 cytoplasmic puncta formation in a TLR9 independent manner. In addition, Src family kinases (SFK), phosphoinositide 3-kinases (PI3K), and DNA-dependent protein kinase (DNA-PK) are known to be activated by the CpG-ODN (16, 17, 18, 19) but inhibition of any of these kinases did not affect the formation of TDP-43 puncta induced by CpG-ODN (Fig. S1, *C*–*F*), suggesting that SFK, PI3K, and DNA-PK are not involved in the CpG-ODN-induced TDP-43 puncta formation.

**Figure 2:**
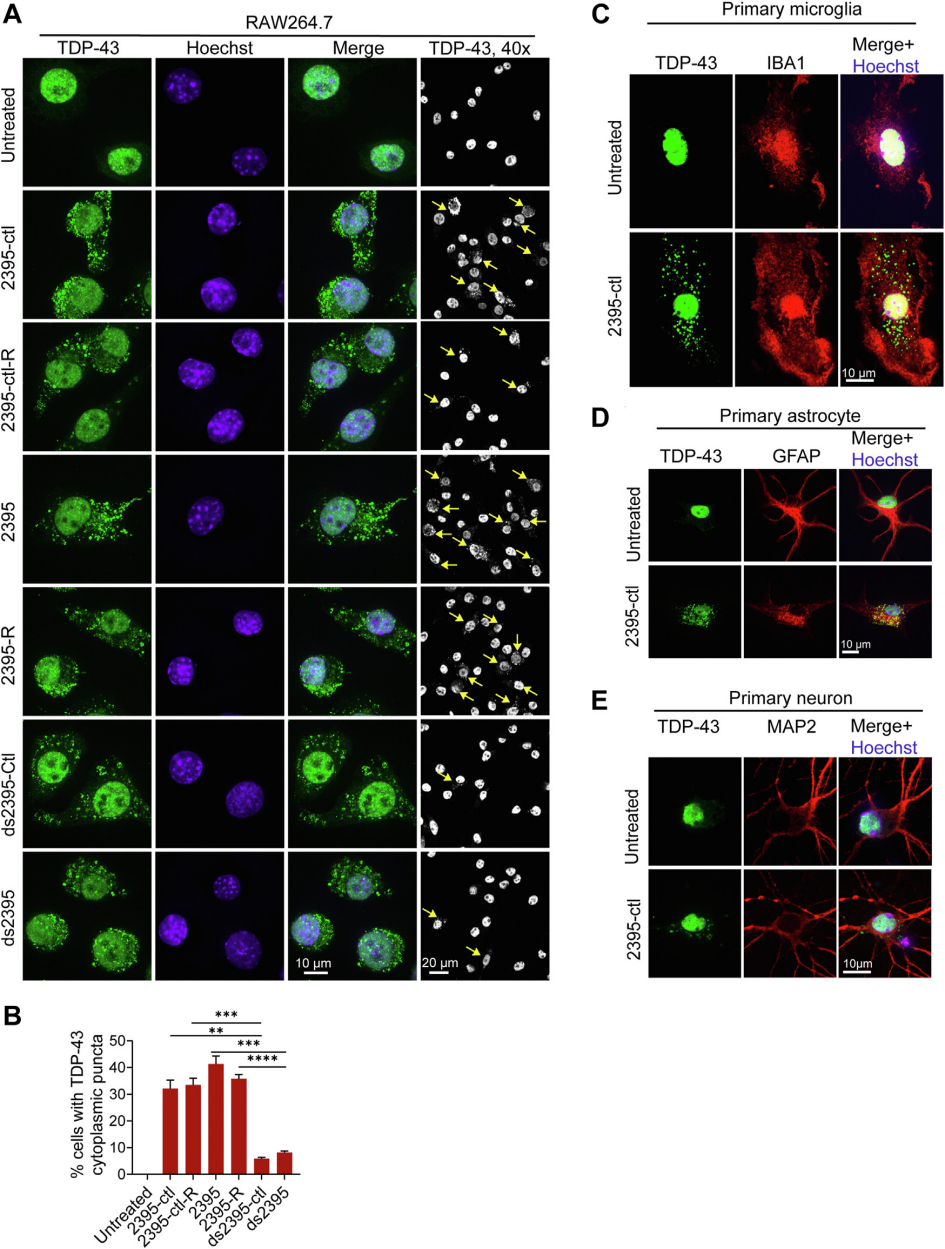
**ODN induces TDP-43 cytoplasmic puncta formation.***A* and *B*, synthetic single- (*A*) and double-stranded (*B*) ODNs induce TDP-43 cytoplasmic puncta formation in the RAW264.7 cells. Cells were treated with the indicated ODNs, and cells were fixed and permeabilized using Triton-X100 after 24 h of treatment. TDP-43 was stained with mouse anti-TDP-43 antibodies. Representative images from three independent experiments were shown for each condition and the 40 x low-magnification images were included (*A*). *Yellow* arrowheads point to the cells with cytoplasmic TDP-43 puncta. Scale bar,10 or 20 μm. The percentage of the cells with cytoplasmic TDP-43 puncta was quantified and 150 to 200 cells from each coverslip were analyzed (*B*). Data are presented as means of ± SEM from 3 independent experiments (n = 3). ∗∗, *p* < 0.01, ∗∗∗, *p* < 0.001, ∗∗∗∗, *p* < 0.0001. Student’s *t* test. (*C*–*E*) ODNs induce TDP-43 cytoplasmic puncta formation in mouse primary microglia (*C*), astrocytes (*D*), and neurons (*E*). Primary cells were treated with 2395-ctl ODN and were permeabilized by Triton-X100. Representative images were shown for TDP-43 and the microglial marker IBA1, astrocyte marker GFAP, or neuronal marker MAP2 staining. Scale bar,10 μm.

Since the CpG-ODN-induced signaling does not seem to participate in the CpG-ODN-induced TDP-43 cytoplasmic puncta formation, we determined the effect of several single-stranded ODNs on the formation of TDP-43 cytoplasmic puncta (Table S1). These ODNs include the negative control ODN for CpG-ODN 2395 (2395-ctl, not containing CpG motif (20)) and the reverse-complementary ODN of 2395-ctl (2395-ctl-R) and 2395 (2395-R). The data showed that all tested ODNs induced the TDP-43 cytoplasmic puncta formation (Fig. 2, *A* and *B*). In addition, we found that double-stranded DNA (dsDNA) can induce the TDP-43 cytoplasmic puncta formation as well (Fig. 2, *A* and *B*). These results suggest that both single- and double-stranded DNA can trigger the cytoplasmic puncta formation of TDP-43. Furthermore, we found that ODN treatment leads to cytoplasmic TDP-43 puncta in primary microglia, astrocytes, and neurons (Fig. 2, *C*–*E*), suggesting ODN-induced TDP-43 puncta formation is not limited to a specific cell type. Considering that macrophage and microglia uptake ODNs more efficiently compared to other cell types, hereafter we used either the macrophage cell line RAW264.7 or primary microglia as cell models to investigate the DNA-induced cytoplasmic TDP-43 puncta. Additionally, to avoid the strong immune activation by the CpG-ODN, the ODN 2395-ctl, which does not have any obvious immunostimulatory activity, was used to induce TDP-43 cytoplasmic puncta formation in the following studies.

### DNA binding promotes liquid-liquid phase separation of TDP-43

**Figure 3:**
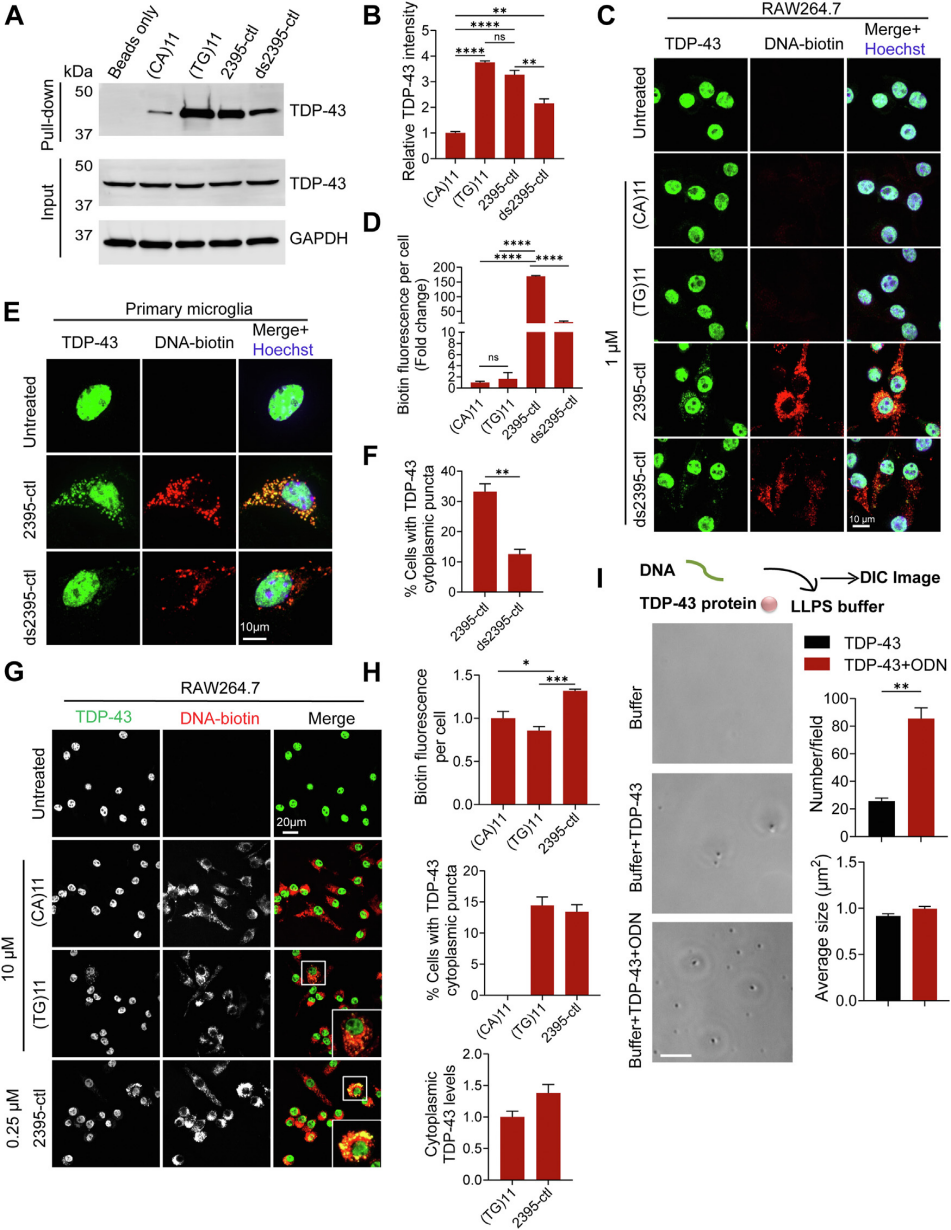
**ODN binds to TDP-43 and facilitates liquid phase separation of TDP-43.***A* and *B*, TDP-43 binds to ODN in *vitro*. RAW264.7 cell lysates were incubated with biotinylated single-stranded ODN (CA)11, (TG)11, 2395-ctl, or double-stranded ODN 2395-ctl (ds2395-ctl), and pull-down experiments were performed by using streptavidin resin. The bound proteins were analyzed by Western blot using the anti-TDP-43 antibodies. The TDP-43 proteins pulled down by the ODN were quantified to compare the binding affinity of TDP-43 to the indicated ODNs (*B*). Data are presented as mean ± SEM from three independent experiments (n = 3). ∗∗, *p* < 0.01, ∗∗∗∗, *p* < 0.0001, ns, not significant, Student’s *t* test. *C* and *D*, TDP-43 colocalizes with ODNs in RAW264.7 cells. RAW264.7 cells were untreated or treated with the indicated biotin-labeled ODNs for 24 h. Cells were fixed and permeabilized using Triton-X100. Representative images show the colocalization between TDP-43 and biotin-ODNs. Scale bar, 10 μm. The biotin fluorescence per cell was quantified from 300 to 400 cells from each coverslip (*D*). Data are presented as means of ± SEM from three independent experiments (n = 3). ∗∗∗∗, *p* < 0.0001. Student’s *t* test. *E and F*, TDP-43 colocalizes with ODNs in primary microglia. Primary microglia were untreated or treated with the indicated biotin-labeled ODNs for 24 h. Cells were fixed and permeabilized using Triton-X100. Representative images show the colocalization between TDP-43 and biotin-ODNs. Scale bar, 10 μm. The percentage of the cells with cytoplasmic TDP-43 puncta was quantified from 150 to 200 cells per coverslip. Data are presented as means of ± SEM from three independent experiments (n = 3). ∗∗, *p* < 0.01, Unpaired two-tailed student’s *t* test. *G* and *H*, uptake of the ODN (TG)11 induces cytoplasmic TDP-43 puncta formation. RAW264.7 cells were untreated or treated with 10 μM of biotin-labeled (CA)11 and (TG)11 and 0.25 μM of 2395-ctl for 24 h. Cells were fixed and permeabilized using Triton-X100. The biotin fluorescence per cell and percentage of the cells with TDP-43 cytoplasmic puncta were quantified from 150 to 200 and 300 to 400 cells from each coverslip, respectively (*H*). The cytoplasmic TDP-43 levels were measured from the cells with TDP-43 cytoplasmic puncta. Data are presented as means of ± SEM from three independent experiments (n = 3). ∗, *p* < 0.05, ∗∗∗, *p* < 0.001. Unpaired two-tailed student’s *t* test. *I*, ODNs promote liquid phase separation of TDP-43. An *in vitro* phase separation assay was performed to determine the effect of DNA on TDP-43 phase separation. Representative DIC images show the TDP-43 liquid droplets formed in the LLPS buffer in the presence or absence of 2395-ctl ODNs. Scale bar, 5 μm. The number and size of the liquid droplets formed in each condition were quantified using Image J. Data are presented as means of ± SEM from three independent experiments (n = 3). ∗∗, *p* < 0.01. Student’s *t* test.

Since TDP-43 can bind to DNA (21, 22), and DNA molecules can mediate the phase separation of the protein (23), we hypothesized that DNA binding to TDP-43 may trigger the formation of TDP-43 cytoplasmic condensates. To test this, we first examined whether TDP-43 can directly interact with ODN. An in *vitro* DNA binding assay was conducted using the biotinylated ODNs. TDP-43 is known to bind to TG-repeated DNA with higher affinity while barely bind to DNA with CA repeats (21, 24, 25). Herein, the TG repeats [(TG)11] and CA repeats [(CA)11] with the same length as 2395-ctl-ODN was included as controls to evaluate the binding ability of 2395-ctl ODN to TDP-43. Single-stranded 2395-ctl ODN showed a slightly lower efficiency compared to (TG)11 but significantly higher efficiency than the double-stranded 2395-ctl ODN, while minimal binding to TDP-43 was observed for (CA)11 ODN (Fig. 3, *A* and *B*). Additionally, deletion analysis confirmed that DNA binding is mediated by the RNA recognition motifs (RRMs) of TDP-43 (Fig. S2), consistent with previous reports that RRMs mediate binding of TDP-43 to DNA (21, 25). Next, we examined the colocalization between TDP-43 and biotin-labeled ODN in the cells after ODN uptake. The ODN (TG)11 and (CA)11 served as the positive and negative control ODNs, respectively. Unfortunately, neither ODN (TG)11 nor (CA)11 were uptaken efficiently to induce TDP-43 cytoplasmic puncta formation when using 1 μM of concentration (Fig. 3, *C* and *D*). We also observed less uptake of the ds2395-ctl by the cells compared to the ss2395-ctl ODNs, while both of them induce TDP-43 puncta and colocalize with TDP-43 in the cytoplasm in RAW264.7 cells (Fig. 3, *C* and *D*) and primary microglia (Fig. 3, *E* and *F*). To further determine that the cytoplasmic TDP-43 puncta formation is mediated by DNA binding, we treated the cells with a higher concentration of (CA)11 and (TG)11 to improve their cellular uptake and examined their ability to induce TDP-43 puncta formation. Efficient uptake of both (CA)11 and (TG)11 were observed when 10 μM of ODNs were added to the culture medium although there was still less uptake compared to the 0.25 μM of 2395-ctl (Fig. 3, *G* and *H*). At 10 μM concentration, ODN (TG)11 but not (CA)11 induces the formation of cytoplasmic TDP-43 puncta (Fig. 3, *G* and *H*). Collectively, these results suggest that the physical interaction between ODNs and TDP-43 is required for the induction of cytoplasmic TDP-43 puncta after ODN uptake. Due to the poor uptake efficiency of (TG)11 and (CA)11, we excluded them as controls in the subsequent uptake experiments.

To further determine the effect of DNA on TDP-43 phase separation, the ability of recombinant TDP-43 to form liquid droplets in the absence or presence of ODNs was examined under a microscope with differential interference contrast (DIC) (26, 27). More liquid droplets were formed in the presence of the ODNs compared to TDP-43 alone and the size of the liquid droplets was barely affected by ODNs (Fig. 3*I*), indicating that ODNs facilitate the liquid-liquid phase separation of TDP-43 *in vitro*. These data support our hypothesis that interaction with cytoplasmic DNA triggers the liquid phase separation of TDP-43.

### ODN-induced TDP-43 puncta are reversible but do not overlap with stress granules

**Figure 4:**
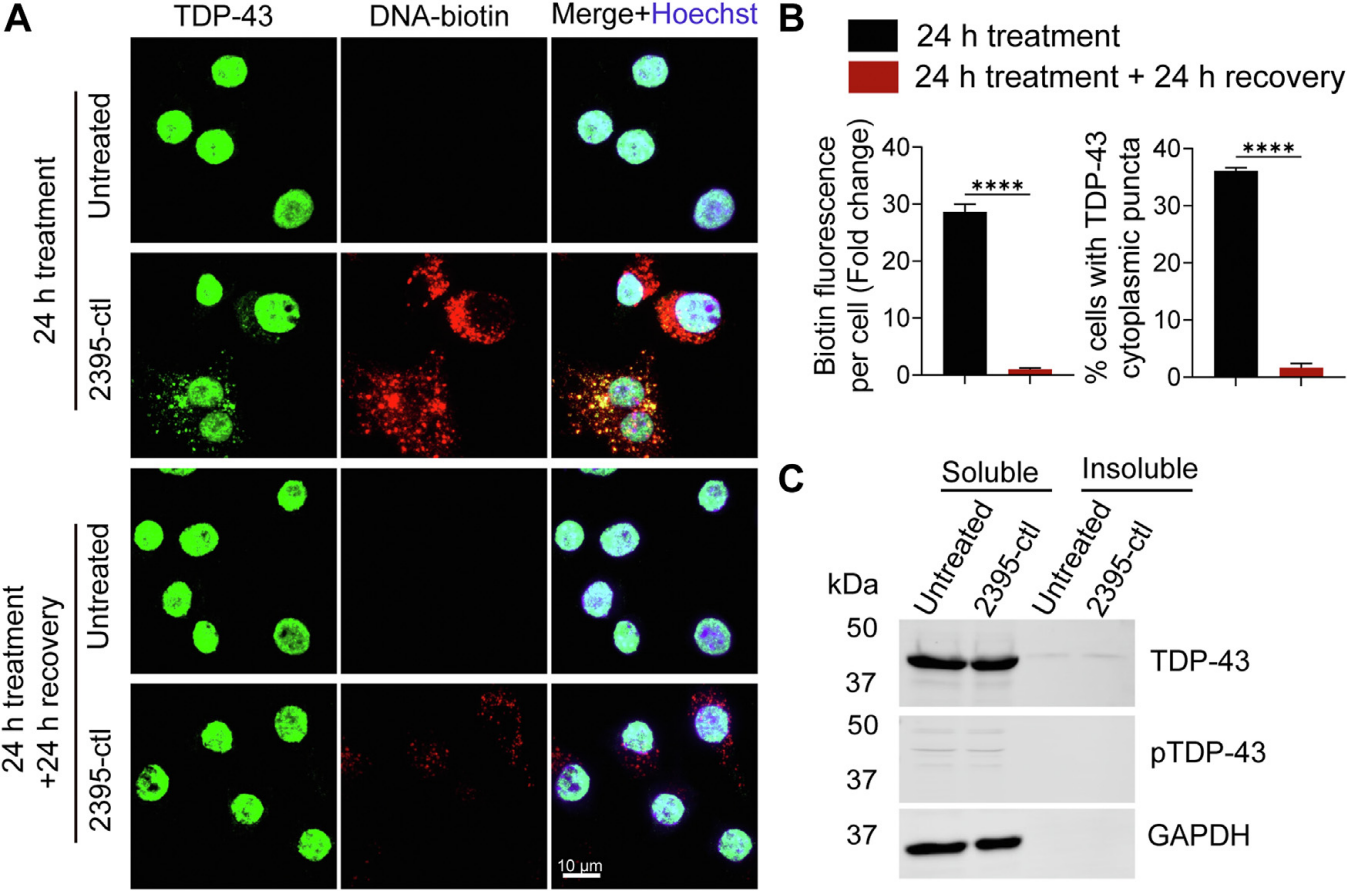
**ODN-induced TDP-43 puncta are reversible.***A* and *B*, ODN-induced cytoplasmic TDP-43 puncta are reversible. RAW264.7 cells were treated with ODN-2395-ctl for 24 h, or the ODNs were removed from the media after 24 h of treatment, and the cells were cultured for another 24 h. Cells were fixed and permeabilized using Triton-X100. TDP-43 distribution was examined, and the percentage of cells with TDP-43 cytoplasmic puncta and the biotin fluorescence per cell was quantified from 100 to 150 cells for each coverslip (*B*). Data are presented as means of ± SEM from three independent experiments (n = 3). ∗∗∗∗, *p* < 0.0001, Student’s *t* test. Scale bar, 10 μm. *C*, the ODN-induced TDP-43 cytoplasmic puncta remain soluble. RAW264.7 cells were treated with the ODN-2395-ctl for 48 h and western blots were performed to analyze the TDP-43 levels and phosphorylation in the RIPA soluble and insoluble fraction. GAPDH was used as a loading control.

**Figure 5:**
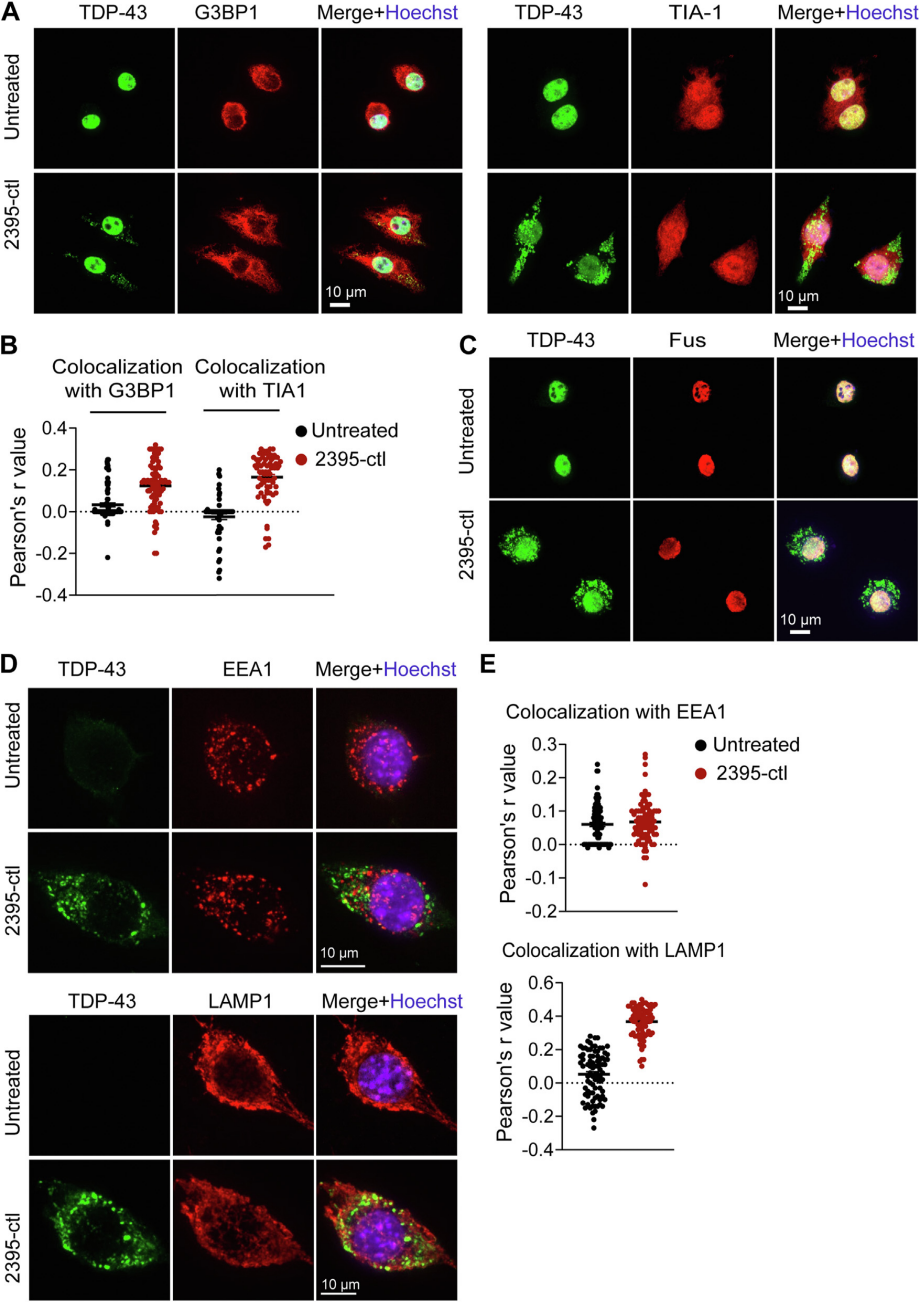
**ODN-induced TDP-43 puncta are not stress granules.***A*–*C*, ODNs-induced TDP-43 cytoplasmic puncta are not stress granules. RAW264.7 cells were *left* untreated or treated with ODN 2395-ctl for 24 h. Cells were fixed and permeabilized using Triton-X100. The colocalization between TDP-43 and the stress granule markers G3BP1, TIA-1, or FUS was examined. Representative images were shown for TDP-43 and G3BP1, TIA-1, or FUS staining. Scale bar,10 μm. The colocalization between TDP-43 and G3BP1 or TIA1 in the cytoplasm was quantified from 60 to 90 cells from three independent experiments. A scatter plot was shown for Pearson’s r value (*B*). *D* and *E*, RAW264.7 cells were left untreated or treated with 2395-ctl for 24 h. Cells were fixed and permeabilized using saponin. The colocalization between TDP-43 and the endosome marker EEA1 or lysosome marker LAMP1 was examined. Scale bar,10 μm. The colocalization between TDP-43 and EEA1 or LAMP1 in the cytoplasm was quantified from 80 to 90 cells from three independent experiments. A scatter plot was shown for Pearson’s r value (*E*).

To investigate the dynamics of ODN-induced TDP-43 punta, we first determined whether ODN-induced TDP-43 punta are reversible after removal of the ODNs. The cells were treated with biotinylated ODNs and then washed to remove ODNs in the extracellular space before incubating for another 24 h. TDP-43 localization pattern, as well as the intracellular ODN levels, were examined. We found that the removal of ODNs leads to a drastic reduction in intracellular ODN levels after 24 h. This was accompanied by the disappearance of cytosolic TDP-43 puncta (Fig. 4, *A* and *B*), indicating the ODN-induced TDP-43 cytoplasmic puncta is reversible. In addition, Western blot analysis of the RIPA soluble and urea soluble fractions showed that ODN-induced TDP-43 cytoplasmic puncta remain soluble and no phosphorylated TDP-43 was detected in both RIPA and urea soluble fractions (Fig. 4*C*), suggesting that the ODN-induced TDP-43 puncta does not mimic the insoluble aggregates seen in the disease state. Since TDP-43 can be recruited into stress granules (SGs) (28), which are dynamic cytosolic condensates formed by the phase separation of RNA molecules and proteins, such as Ras GTPase-activating protein-binding protein 1 (G3BP1) and cytotoxic granule-associated RNA binding protein (TIA1), we wondered whether the ODN-induced TDP-43 cytoplasmic puncta are SGs. We found ODN treatment did not induce visible cytosolic granules positive with G3BP1 or TIA1, and G3BP1 or TIA-1 did not localize to ODN-induced TDP-43 puncta (Fig. 5, *A* and *B*), suggesting that the ODN-induced TDP-43 cytoplasmic puncta are not stress granules. Moreover, ODN treatment did not affect the cellular distribution of the fused in sarcoma (FUS) protein, a DNA/RNA binding protein that has similar functions with TDP-43 (29, 30) (Fig. 5*C*). Previous studies have suggested that exogenous DNA oligonucleotides, including CpG-ODN and nonstimulatory ODNs, uptaken by the cells can enter endosomes or lysosomes (31, 32, 33). Thus, we examined whether the ODN-induced TDP-43 puncta are localized in these compartments, and we found that the TDP-43 puncta were mostly localized in the cytosol instead of in EEA1-positive endosomes or LAMP1-positive lysosomes (Fig. 5, *D* and *E*).

### Prolonged cytoplasmic DNA accumulation leads to nuclear depletion of TDP-43 and generation of a TDP-43 short isoform or fragment

**Figure 6:**
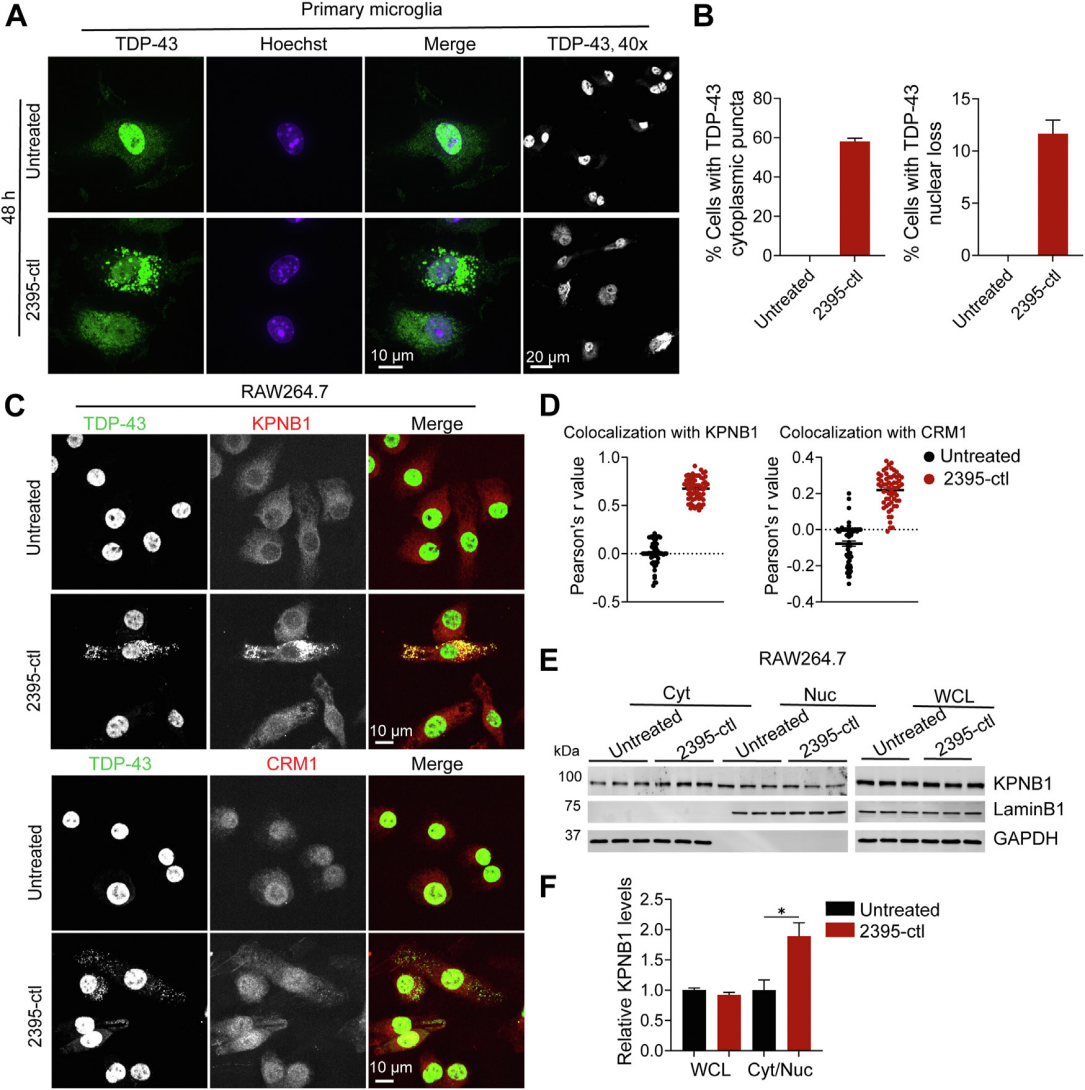
**Persistent ODN accumulation in cytosol causes nuclear depletion of TDP-43.***A* and *B*, ODN treatment leads to the cytoplasmic accumulation of TDP-43 in primary microglia. Primary microglia were treated with ODN 2395-ctl for 48 h. Cells were fixed and permeabilized using Triton-X100. Representative images were shown for TDP-43 distribution and the 40 x low magnification images were included (*A*). Scale bar, 10 μm. The percentage of the cells with cytoplasmic TDP-43 puncta or cells showing TDP-43 nuclear loss was quantified from 240 to 327 cells for each coverslip (B). Data are presented as means of ± SEM from three independent experiments (n = 3). *C* and *D*, KPNB1 colocalizes with TDP-43 in the cytoplasm. RAW264.7 cells were left untreated or treated with ODN 2395-ctl for 24 h. Cells were fixed and permeabilized using Triton-X100. Representative images were shown for TDP-43, KPNB1, and CRM1 distribution. Scale bar, 10 μm. The colocalization between TDP-43 and KPNB1 or CRM1 in the cytoplasm was quantified from 60 cells from three independent experiments. A scatter plot was shown for Pearson’s r value (*D*). *E* and *F*, cytoplasmic and nuclear levels of KPNB1 and CRM1 in the 2395-ctl ODN-treated cells. Cells were treated as above and subjected to a nuclear and cytoplasmic fractionation. The levels of the indicated proteins in nuclear (Nuc) and cytoplasmic (Cyt) fractions and whole-cell lysates (WCL) were analyzed by Western blot (*E*). LaminB1 was used as a nuclear loading control and marker and GAPDH as a cytosolic loading control and marker. The band intensities were measured by ImageJ and the relative KPNB1 levels in WCL and the ratio of the KPNB1 levels in cytoplasm and nucleus (Cyt/Nuc) were quantified (*F*). Data are presented as means of ± SEM from three independent experiments (n = 3). ∗, *p* < 0.05. Student’s *t* test.

TDP-43 proteinopathy in neurodegenerative diseases is typically characterized by cytoplasmic aggregation and nuclear loss of TDP-43. We found that prolonged DNA treatment leads to not only the accumulation of TDP-43 in the cytoplasm but also a significant reduction of nuclear TDP-43 in primary microglia (Fig. 6, *A* and *B*), suggesting that persistent DNA-induced cytoplasmic TDP-43 puncta formation leads to the mislocalization of TDP-43 and potentially loss of function of TDP-43 in the nucleus. Given that impaired nucleocytoplasmic transport can lead to mislocalization of TDP-43 (10, 34), we asked whether uptake of the exogenous ODN could affect nucleocytoplasmic transport. The cytoplasmic and nuclear distribution of the nuclear import receptor karyopherin subunit β1 (KPNB1, also known as importin β1) and the nuclear export receptor chromosomal maintenance 1 (CRM1, also known as exportin 1) were examined in the ODNs-treated cells. Interestingly, a strong colocalization between TDP-43 and KPNB1 but not CRM1 was observed in the cytoplasm in ODNs-treated cells (Fig. 6, *C* and *D*). Additionally, an increase in the cytoplasmic KPNB1 levels and a decrease in its nuclear levels were observed in ODN-treated cells (Fig. 6, *E* and *F*), suggesting the ODNs accumulation leads to the sequestration of KPNB1 in TDP-43 puncta and the retention of KPNB1 in the cytoplasm, which might further impair TDP-43 nuclear import and eventually lead to nuclear loss of TDP-43.

**Figure 7:**
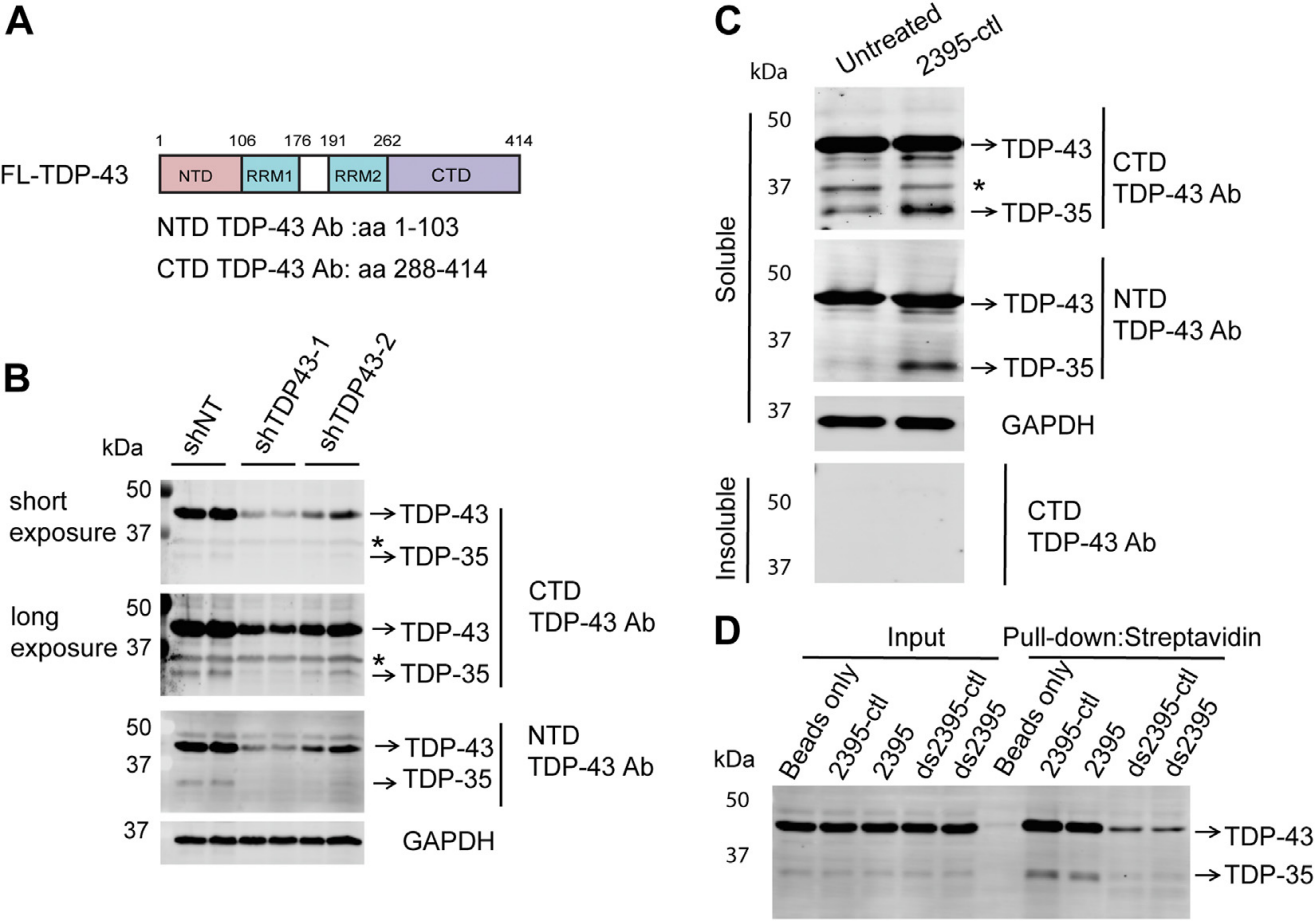
**ODN accumulation in cytosol leads to increased levels of TDP-35.***A*, domain structure of full-length TDP-43. NTD: N-terminal domain, RRM: RNA recognition motif, CTD: C-terminal domain. *B*, the short TDP-43 species can be knocked down by shRNA targeting TDP-43. RAW264.7 cells were transduced with TDP-43 shRNA (shTDP-43–1 and shTDP-43–2) lentivirus for 2 to 3 days and TDP-43 levels were detected by Western blot using anti-TDP-43 CTD rabbit and NTD mouse antibodies. ∗: non-specific band detected by the anti-TDP-43 CTD rabbit antibodies. *C*, ODN treatment increases TDP-35 levels in primary microglia. Primary microglia were treated with ODN for 48 h and the protein levels of TDP-43 in RIPA soluble and insoluble fractions were detected by using anti-TDP-43 CTD rabbit and NTD mouse antibodies. ∗: non-specific band detected by the anti-TDP-43 CTD rabbit antibodies. *D*, TDP-35 binds to ODN in *vitro*. RAW264.7 cell lysates were incubated with biotinylated ODNs, and pull-down experiments were performed by using streptavidin resin. The bound proteins were analyzed by Western blot using the anti-TDP-43 NTD mouse antibodies.

Accumulation of short TDP-43 isoforms and fragments, generated through the translation of alternatively spliced isoforms or proteolytic cleavage, is a hallmark of TDP-43 pathology (35, 36, 37). We observed the presence of a ∼35 kDa band of TDP-43 in RAW264.7 cell lysates, which can be detected by antibodies raised against NTD (amino acid 1–103) and CTD (amino acid 288–414) of TDP-43 (Fig. 7, *A* and *B*). The intensity of this band decreased when TDP-43 was knocked down by the shRNAs targeting exon 2 or 5 of *Tardbp* (Fig. 7*B*), indicating that this low molecular weight of TDP-43 species, denoted here as TDP-35, might be a splice variant or cleaved fragment of TDP-43. Interestingly, the levels of TDP-35 were increased upon ODN treatment (Fig. 7*C*). Several TDP-43 variants or fragments display reduced solubility and promote TDP-43 aggregation (38, 39). However, neither full-length TDP-43 nor TDP-35 was detected in the insoluble fraction from the untreated or ODN-treated cells (Fig. 7*C*), suggesting the ODNs may not affect the solubility of TDP-35. Additionally, streptavidin beads pull-down of biotinylated ODNs showed that the TDP-35 can bind to the ODN in *vitro* (Fig. 7*D*), but it remains to be tested whether it could interact with ODN in the cell, as we do not have antibodies specifically recognizing this form of TDP-43. Nevertheless, our findings demonstrate that persistent DNA uptake leads to TDP-43 mislocalization and accumulation of shortened TDP-43 species.

### TDP-43 Q331K impairs the disassembly of cytoplasmic TDP-43 puncta induced by DNA accumulation

**Figure 8:**
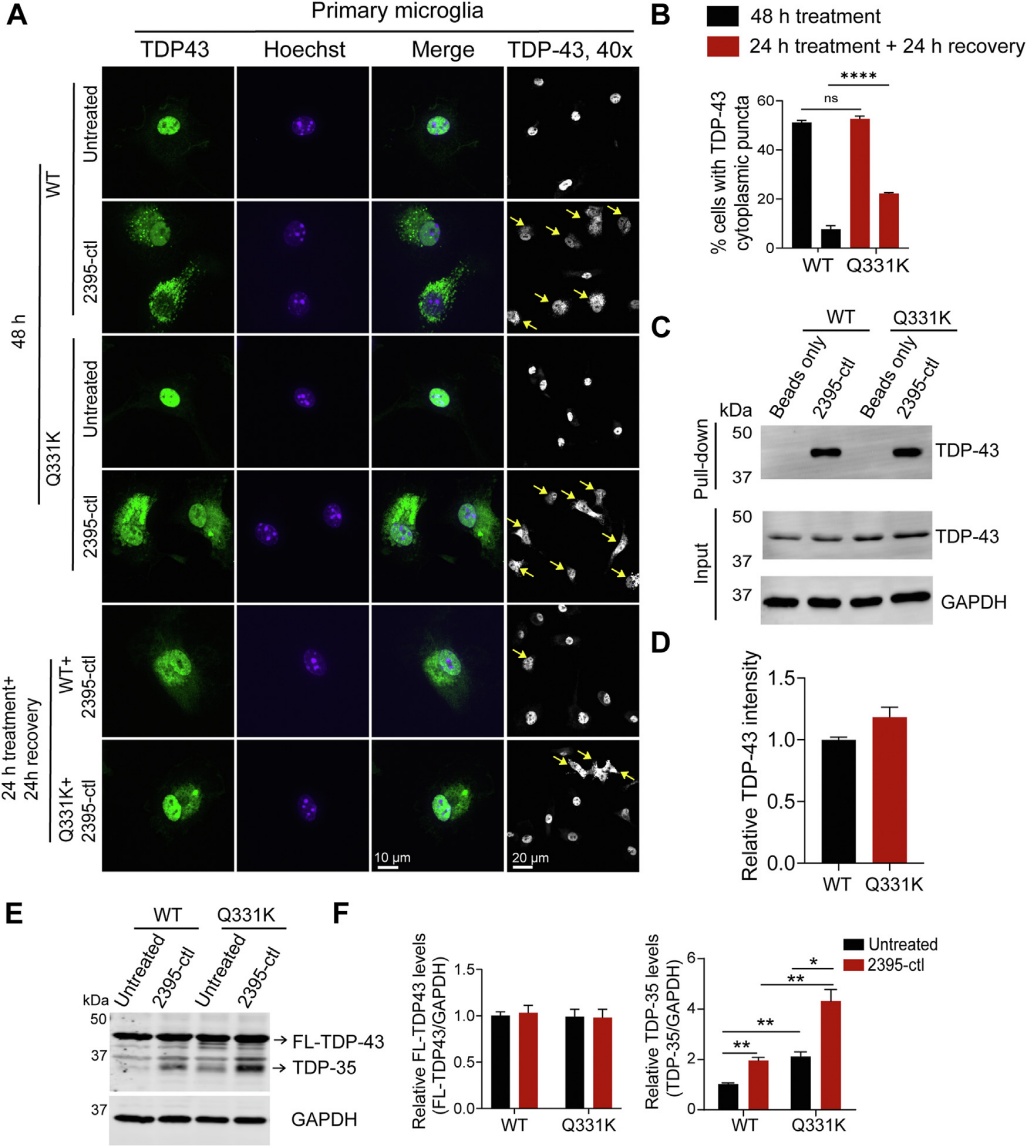
**Q331K mutation impairs the turnover of the ODN-induced cytoplasmic TDP-43 puncta.***A* and *B*, Q331K mutation renders the DNA-induced TDP-43 puncta more irreversible. WT and *TDP-43*^*Q331K*^ primary microglia were treated with ODN for 48 h, or the ODNs were washed out after 24 h of treatment, and cells were cultured for another 24 h. Cells were fixed and permeabilized using Triton-X100. Representative images were shown for TDP-43 distribution in the cells and the 40 x low magnification images were included. *Yellow* arrowheads point to the cells with cytoplasmic TDP-43 puncta. Scale bar, 10 μm. The percentage of the cells with TDP-43 cytoplasmic puncta was quantified from 139 to 181 cells for each coverslip (*B*). Data are presented as mean ± SEM from three experiments (n = 3). ∗∗∗∗, *p* < 0.0001, Student’s *t* test. *C* and *D*, WT and *TDP-43*^*Q331K*^ brain lysates were incubated with biotinylated 2395-ctl, and streptavidin resin was used to pull down biotinylated ODN and its associated proteins. The bound proteins were analyzed by Western blot using the anti-TDP-43 antibodies. The TDP-43 proteins pulled down by the ODNs were quantified to compare the binding affinity of TDP-43 to 2395-ctl ODNs (D). Data are presented as mean ± SEM from three independent experiments (n = 3). Student’s *t* test. *E and F*, ODN treatment leads to higher levels of TDP-35 in *TDP-43*^*Q331K*^ microglia. WT and *TDP-43*^*Q331K*^ primary microglia were treated with ODN for 48 h and the protein levels of TDP-43 were measured by Western blot using the anti-TDP-43 rabbit antibodies. GAPDH was used as a loading control. The protein levels of full-length TDP-43 (FL-TDP43) and TDP-35 were quantified and normalized to GAPDH. Data are presented as mean ± SEM from three independent experiments (n = 3). ∗, *p* < 0.05, ∗∗, *p* < 0.01. Student’s *t* test.

The ALS-associated mutation Q331K, located at the prion-like low complexity domain (PrLD) of TDP-43, has been found to regulate the phase separation properties of TDP-43 in *vitro* (40). To determine the effect of Q331K on the formation of DNA-induced TDP-43 puncta, we cultured primary microglia from WT or transgenic mice with *TDP-43*^*Q331K*^ knocked in (41) and treated the cells with ODNs. The percentage of the microglia with cytoplasmic TDP-43 puncta was similar between WT and Q331K cells after 48 h of ODN treatment (Fig. 8, *A* and *B*), suggesting the formation of the ODN-induced TDP43 puncta is not affected by Q331K. However, more cells were found to retain cytoplasmic TDP-43 puncta after the removal of ODNs in *TDP-43*^*Q331K*^ microglia compared to WT microglia (Fig. 8, *A* and *B*), indicating that Q331K mutation delays the resolution of ODN-induced TDP-43 cytoplasmic puncta after ODN degradation. To better understand how TDP-43 Q331K affects the disassembly of cytoplasmic TDP-43 puncta, we determined whether the Q331K mutation alters the interaction between TDP-43 and DNA. Q331K does not have any significant effect on TDP-43 binding to biotinylated ODN 2395-ctrl in the streptavidin pull-down assay (Fig. 8, *C* and *D*). Moreover, Q331K has been shown to perturb TDP-43 alternative splicing (41). We observed increased levels of TDP-35 in primary microglia with Q331K mutation (Fig. 8, *E* and *F*). TDP-35 levels were upregulated in both WT and *TDP-43*^*Q331K*^ microglia upon ODN treatment but the upregulation was more pronounced in *TDP-43*^*Q331K*^ microglia (Fig. 8, *E* and *F*). Taken together, our results support that Q331K impairs the disassembly of DNA-induced TDP-43 cytoplasmic puncta and alters the expression pattern or protein homeostasis of TDP-43, especially in response to the accumulation of cytoplasmic DNA.

### TDP-43 cytoplasmic puncta are present in pathological conditions with cytosolic DNA accumulation

**Figure 9:**
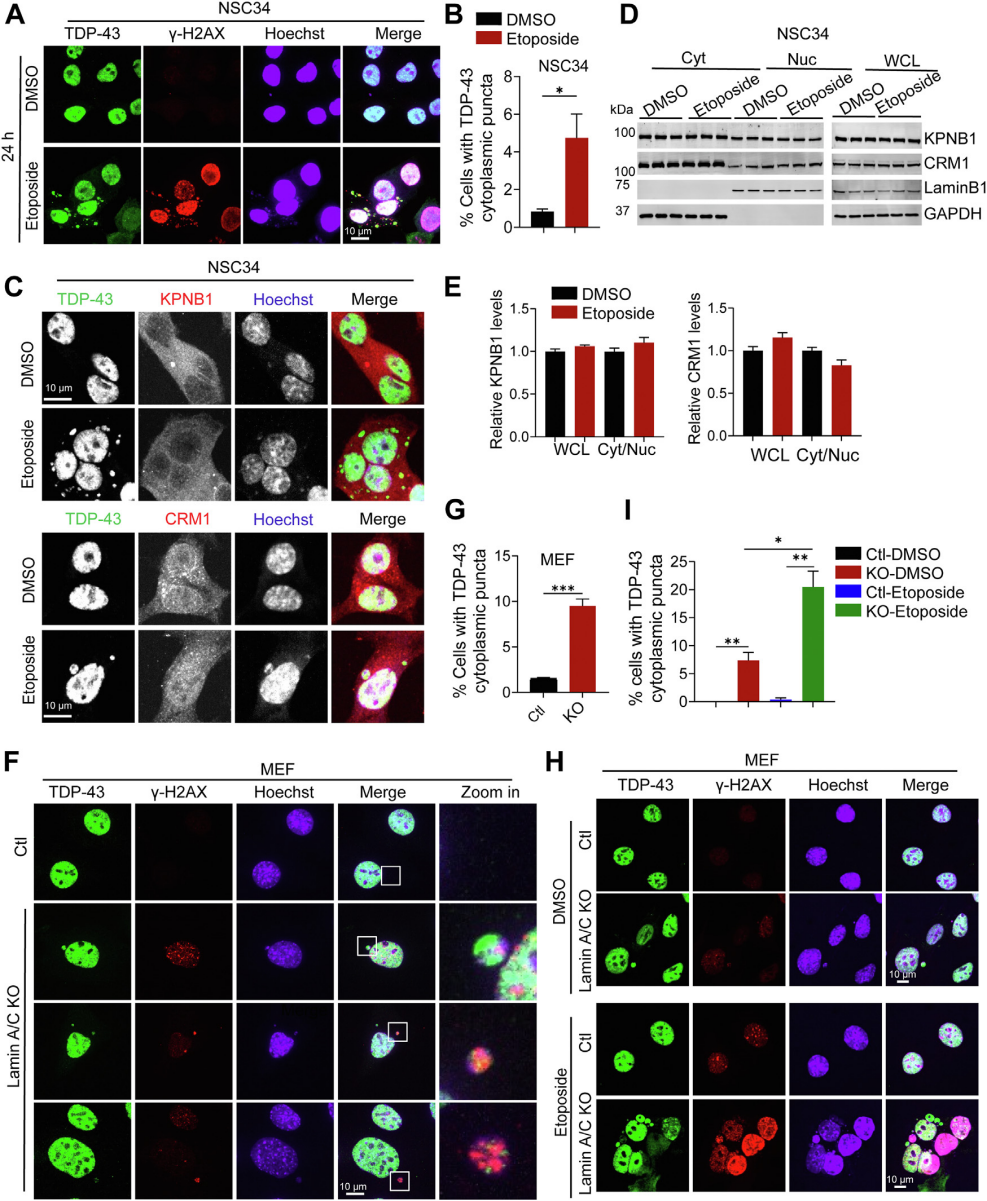
**Induction of TDP-43 cytoplasmic puncta by DNA damage or by Lamin A/C deficiency.***A* and *B*, DNA damage inducer etoposide triggers the formation of TDP-43 puncta in NSC34 cells. Cells were treated with 5 μM etoposide or DMSO for 24 h. TDP-43 distribution was detected by immunostaining after cells were fixed and permeabilized using Triton X-100. Representative images were shown for TDP-43 and the DNA damage marker γ-H2AX. Scale bar, 10 μm. The percentage of the cells with cytoplasmic TDP-43 accumulation was quantified (*B*). Data are presented as means of ± SEM from three independent experiments (n = 3). ∗, *p* < 0.05. Student’s *t* test. *C*, distribution of the KPNB1 and CRM1 in the etoposide-treated NSC34 cells. Cells were treated with 5 μM etoposide or DMSO for 24 h. TDP-43 distribution was detected by immunostaining after cells were fixed and permeabilized using Triton X-100. Representative images were shown for TDP-43 and KPNB1 or CRM1. Scale bar, 10 μm. *D*, cytoplasmic and nuclear levels of KPNB1 and CRM1 in the etoposide-treated NSC34 cells. Cells were treated as above and subjected to nuclear and cytoplasmic fractionation. The levels of the indicated proteins in nuclear (Nuc) and cytoplasmic (Cyt) fractions and whole-cell lysates (WCL) were analyzed by Western blot (*E*). LaminB1 was used as a nuclear loading control and marker and GAPDH as a cytosolic loading control and marker. The band intensities were measured by ImageJ, and the relative KPNB1 and CRM1 levels in WCL and the ratio of their levels in cytoplasm and nucleus (Cyt/Nuc) were quantified (*E*). Data are presented as means of ± SEM from three independent experiments (n = 3). Student’s *t* test. *F* and *G*, lamin A/C deficiency leads to cytoplasmic TDP-43 puncta formation. WT control and Lamin A/C-deficient MEF cells were stained with TDP-43 and γ-H2AX. The percentage of the cells with cytoplasmic TDP-43 puncta was quantified (*G*). Data are presented as means of ± SEM from three independent experiments (n = 3). ∗∗∗, *p* < 0.001. Student’s *t* test. *H* and *I*, DNA damage leads to increased cytoplasmic TDP-43 accumulation in Lamin A/C-deficient cells. WT control and Lamin A/C-deficient MEF cells were treated with 5 μM etoposide or DMSO for 24 h. Representative images were shown for the TDP-43 and γ-H2AX. The percentage of cells with cytoplasmic TDP-43 puncta in each condition was quantified (*I*). Data are presented as means of ± SEM from 3 independent experiments (n = 3). ∗, *p* < 0.05, ∗∗, *p* < 0.01. Student’s *t* test.

So far, our data show that synthesized DNA oligos uptaken by the cell triggers the formation of cytoplasmic TDP-43 condensates. Next, we asked whether the cytosolic accumulation of endogenous DNA under disease conditions could trigger TDP-43 pathology. While DNA is restricted to the nucleus and mitochondria under normal conditions, endogenous cytosolic DNA species containing genomic DNA, such as micronuclei (MN) and cytoplasmic chromatin fragments (CCFs), have been found in multiple pathological conditions, including DNA damage and nuclear envelope dysfunction (42, 43). To determine whether DNA damage induces cytoplasmic TDP-43 puncta formation, we treated the cells with etoposide, a DNA-damaging agent. Phosphorylation of H2AX at Ser 139 (γ-H2AX) is a commonly used marker for DNA damage (44, 45). γ-H2AX levels were significantly increased in etoposide-treated motor neuron cell line NSC34 cells compared to the DMSO-treated controls (Fig. 9*A*), confirming the induction of DNA damage. We found that etoposide treatment induces TDP-43 cytoplasmic puncta in cells with DNA damage. In some cells, TDP-43 puncta appears to be within the MN or CCFs-like structures labeled by the DNA dye Hoechst and the marker for cytoplasmic MN or CCFs, γH2AX (43) (Fig. 9, *A* and *B*). The cytoplasmic and nuclear distributions and levels of KPNB1 and CRM1 were not affected by etoposide treatment (Fig. 9, *C*–*E*), suggesting that the cytoplasmic TDP-43 accumulation in the etoposide-treated cells is not caused by the impairment of nucleocytoplasmic transport.

Deficiency of nuclear intermediate filament component lamins has been shown to cause cytosolic DNA accumulation due to loss of nuclear envelope integrity (43, 46). In line with this, MN and CCFs can be observed in cells lacking Lamin A/C (47, 48), which can be stained with Hoechst and γH2AX antibodies (Fig. 9*F*). Meanwhile, punctate TDP-43 signals were found in these structures (Fig. 9, *F* and *G*). Additionally, treatment with etoposide results in increased accumulation of cytoplasmic TDP-43 puncta in Lamin A/C-deficient cells (Fig. 9, *H* and *I*). Taken together, these observations support that cytoplasmic DNA accumulation caused by DNA damage or disrupted nuclear envelope integrity may be one potential inducer of cytoplasmic TDP-43 accumulation. Intriguingly, a recent study has identified pathogenic somatic mutations in *LMNA*, the gene encoding Lamin A/C, in sporadic ALS patients (49). The presence of cytosolic TDP-43 pathology in Lamin A/C deficient cells as revealed in our study might provide mechanistic insights to the link between *LMNA* mutations and ALS.

## Discussion

TDP-43 proteinopathy is widely associated with neurodegenerative disorders. However, the upstream events leading to TDP-43 aggregation remain largely unknown. In this study, we demonstrate that the exogenous DNA molecules uptaken by the cells induce cytosolic TDP-43 condensates through direct binding to TDP-43. TDP-43 is mislocalized from the nucleus to the cytoplasm with persistent cytosolic DNA accumulation. In addition, TDP-43 cytoplasmic puncta are induced by DNA damage or compromised nuclear envelope integrity, which leads to the accumulation of cytosolic DNA. Our studies reveal the novel role of DNA binding in regulating TDP-43 proteostasis and provide new insights into the molecular mechanism underlying TDP-43 pathology.

### The origins of cytosolic DNA molecules

**Figure 10:**
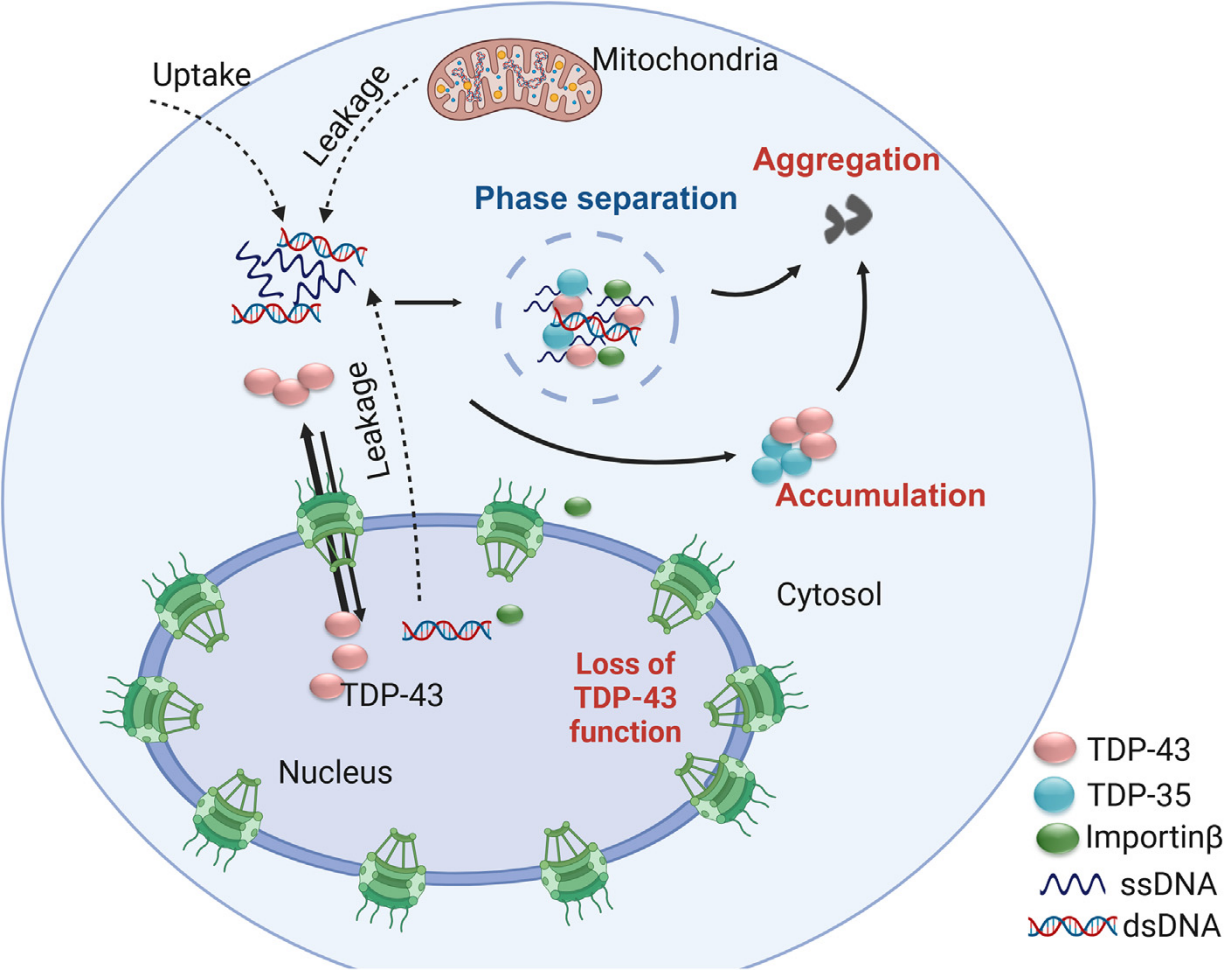
**Proposed model for the role of DNA-induced TDP-43 cytoplasmic puncta formation in TDP-43 pathology.** In healthy cells, TDP43 normally shuttles between the nucleus and cytoplasm, keeping proper cytoplasmic and nuclear levels. Under disease conditions, abnormal DNA accumulation in the cytoplasm, due to leakage of DNA from the nucleus or mitochondria or uptake of DNA from the extracellular space, induces the formation of cytosolic TDP-43 condensates, leading to the sequestration of TDP-43 and the nuclear import receptor KPNB1 in the cytoplasm and the reduction of nuclear TDP-43 levels. Cytosolic DNA accumulation also leads to an increase in the levels of a short TDP-43 isoform, TDP-35. TDP-35 might further sequester full-length TDP-43 to form the pathological aggregates in the cytoplasm. Thus, DNA-induced TDP-43 cytoplasmic puncta formation might be a critical step in seeding TDP-43 aggregation in both neurons and glial cells (Figure created in https://BioRender.com).

Herein, we show that the accumulation of cytoplasmic DNA may be involved in TDP-43 aggregation during neurodegeneration. Endogenous cytoplasmic DNA species, such as micronuclei (MN) and cytoplasmic chromatin fragments (CCFs), can be generated under disease conditions, including DNA damage and nuclear membrane disruption (43, 48). There is growing evidence that DNA damage and loss of nuclear envelope integrity are implicated in aging and neurodegeneration (50, 51, 52, 53, 54, 55), and DNA damage has been linked to TDP-43 proteinopathies, such as in ALS (51, 56). In addition, these two events might coexist and reinforce each other under the same disease condition, as DNA damage can induce nuclear envelope rupture (57) and nuclear envelope rupture leads to a further increase in DNA damage according to previous studies (55, 58, 59). We found that etoposide-induced DNA damage leads to TDP-43 accumulation in the cytoplasm, which is exacerbated under Lamin A/C- deficient conditions (Fig. 9). Lamins A and C, are nuclear intermediate-filament proteins found in nearly all somatic cells, and mutations in the Lamins A/C gene *LMNA* are linked to a variety of diseases affecting striated muscle, adipocytes, or peripheral nerves or causing premature aging (46). Although it is unknown whether Lamin A/C deficiency directly causes TDP-43 pathology, a recent study has identified pathogenic somatic mutations in *LMNA*, the gene encoding Lamin A/C, in sporadic ALS patients (49). The formation of TDP-43 cytoplasmic puncta in etoposide-treated cells, Lamin A/C deficient cells as well as in the cells uptaken exogenous ODNs all support a key role of cytosolic DNA accumulation in inducing cytosolic TDP-43 puncta. Since DNA damage is closely tied to neurodegenerative diseases as well as aging (50, 51), the DNA released from the nucleus to cytosol upon DNA damage or impaired nucleus integrity during aging and neurodegeneration could be one of the mechanisms inducing TDP-43 cytoplasmic puncta formation (Fig. 10). Further characterization of the TDP-43 cytoplasmic puncta and dissecting the precise mechanisms leading to the formation under these conditions will lead to a better understanding on the role of abnormal DNA accumulation in TDP-43 pathology.

In addition to the DNA released from nucleus, cytosolic DNA can also be released by mitochondria as a result of mitochondrial dysfunction or impaired mitophagy (60, 61) (Fig. 10). Since the DNA fragments derived from mtDNA (mt6160) can induce the TDP-43 puncta formation (Fig. S4) and mitochondrial damage has been associated with multiple neurodegenerative diseases, including ALS (61), the cytosolic DNA generated from damaged mitochondria might induce the TDP-43 cytoplasmic puncta. Moreover, DNA molecules produced in the above conditions can get released to the extracellular space during cell death (62, 63) and get uptaken by microglia or astrocytes in the brain and trigger TDP-43 cytoplasmic puncta in these cells (Fig. 10). Although TDP-43 cytoplasmic aggregation is mainly seen in neurons, it can also be found in glial cells, including microglia and astrocytes (13, 64, 65). Our studies from cell culture support that the abnormally accumulated cytoplasmic DNA may be one of the mechanisms leading to TDP-43 mislocalization likely in both neurons and glial cells.

ODNs are believed to be endocytosed by cells followed by intracellular trafficking into endosomes and lysosomes (66). However, ODNs can escape from these compartments and be released to the cytosol though the underlying mechanisms are not well understood (66, 67, 68). Our findings indicate that ODN-induced TDP-43 puncta are primarily localized in cytosol rather than in lysosomes or endosomes (Fig. 5, *D* and *E*). As TDP-43 signals do partially overlay with LAMP1 and TDP-43 contains a chaperone-mediated autophagy (CMA)-recognition motif that allows its entry and degradation *via* lysosomes (69), TDP-43 and ODN interactions could potentially happen in lysosomes and the ODN-induced TDP-43 puncta might be degraded by lysosomes and affected by lysosomal dysfunction. Future work is needed to investigate this possibility.

### DNA binding ability of TDP-43

TDP-43 can bind both ssDNA and dsDNA (22). In line with this, we have found that TDP-43 binds to both ss and dsODNs with a much higher affinity to the ssODN (Fig. 3, *A* and *B*). As a consequence of this and the higher uptake efficiency of ssDNA compared to dsDNA (Fig. 3, *C* and *D*), the ssODN induces more cytoplasmic TDP-43 puncta in the cells (Figs. 2, *A* and *B*; 3, *E* and *F*). Studies have shown that TDP-43 prefers to bind to the TG-enriched ssDNA (22). Unexpectedly, the 2395-Ctl ODN we used shows comparable binding affinity to the TDP-43 compared to the TG-enriched ODN (Fig. 3, *A* and *B*). However, (TG)11 ODNs can not be efficiently taken up by the cell, and as a result, they do not induce TDP-43 cytoplasmic puncta formation unless a higher concentration is used (Fig. 3, *C*, *D*, *G* and *H*). In contrast, the (CA)11 ODNs, which only weakly bind to TDP-43 (Fig. 3*A*), do not induce TDP-43 cytoplasmic puncta even under conditions with cellular uptake comparable with the (TG)11 ODNs (Fig. 3, *C*, *D*, *G* and *H*). Taken together, these data demonstrate that the direct DNA-TDP-43 binding mediates TDP-43 cytoplasmic puncta formation in the cell and cytoplasmic TDP-43 puncta formation is dependent on the concentration of cytosolic DNA as well as the binding affinity of cytosolic DNA to TDP-43.

### Regulation of TDP-43 phase separation properties by DNA

TDP-43 can undergo phase transitions, including condensation into liquid droplets and accumulation into irreversible aggregates (6, 7). Under physiological conditions, TDP-43 can form liquid-like droplets through liquid-liquid phase separation (LLPS), retaining TDP-43 in its functional form in the highly dynamic state. The interaction with the nucleic acids has been shown to modulate TDP-43 liquid-liquid phase separation (70). Our studies show that DNA-induced TDP-43 puncta is reversible in the cell (Fig. 4, *A* and *B*) and DNA molecules enhance the formation of TDP-43 liquid droplets in *vitro* (Fig. 3*I*). Hence, the cytoplasmic TDP-43 puncta are likely condensates formed by DNA-mediated liquid phase separation of TDP-43. Under physiological conditions, TDP-43 exists in a dispersed state in the cytosol with relatively low levels. When DNA fragments accumulate in the cytoplasm, DNA molecules interact with TDP-43 to drive phase transitions of TDP-43, which are reversible upon the removal of the DNA molecules (Fig. 4, *A* and *B*). However, persistent ODN accumulation might change the liquid-like properties, making it irreversible over time and also leading to the depletion of nuclear TDP-43.

### The effect of Q331K mutation on DNA-induced phase separation of TDP-43

The ALS-associated mutations, especially those located in the intrinsically disordered prion-like domain of TDP-43, have been shown to alter the liquid phase separation of TDP-43 by disrupting the TDP-43 intermolecular CTD interactions (7, 71, 72, 73). Q331K is one such mutation, but it remains controversial regarding its effect on TDP-43 phase separation. One of the previous studies has revealed that ssDNA triggers the formation of irreversible aggregates of Q331K mutant protein in *vitro* (74). Consistent with this, we have found that the Q331K mutation impairs the disassembly of the DNA-induced cytoplasmic TDP-43 condensates after the removal of the ODNs (Fig. 8, *A* and *B*), suggesting that the liquid-like properties of TDP-43 might be impaired by Q331K. These findings reveal a link between ALS-related TDP-43 mutations and aberrant phase transition of TDP-43. Furthermore, we found that Q331K mutation does not affect TDP-43 binding to ODNs (Fig. 8, *C* and *D*). It therefore remains to be determined how Q331K affects the TDP-43 phase transition in response to cytosolic DNA accumulation.

### Regulation of TDP-43 homeostasis by DNA

The nucleocytoplasmic shuttling of TDP-43 must be tightly regulated to maintain physiological levels of TDP-43 in the nucleus, which is essential for its cellular functions. With the persistent presence of DNA molecules in the cytosol, continuous interaction of cytoplasmic TDP-43 with DNA leads to the loss of nuclear TDP-43 (Fig. 6). Although no insoluble and phosphorylated TDP-43 was detected in the cells with TDP-43 cytoplasmic mislocalization (Figs. 4*C*, 7*C*), it is plausible that DNA-induced cytoplasmic puncta formation represents an early event preceding TDP-43 aggregation under pathological conditions. It is well documented that dysfunction in nucleocytoplasmic transport is involved in TDP-43 pathology (10, 34, 75, 76). TDP-43 is imported into the nucleus *via* the karyopherin-α proteins (KPNAs) and KPNB1-mediated pathway, while the CRM1-mediated pathway is one of the mechanisms for TDP-43 nuclear export (10). Interestingly, the ODNs-induced TDP-43 puncta sequester the nuclear import receptor KPNB1 but not the nuclear export receptor CRM1 in the cytoplasm (Fig. 6, *C*–*F*), which in line with the previous findings that KPNB1 is recruited into TDP-43 aggregates in cell models as well as in ALS/FTD patients (77). The sequesteration of karyopherins and nuclear pore proteins in the TDP-43 aggregates contributes to clearance of nuclear TDP-43 due to impaired nucleocytoplasmic transport (78). These findings indicate that aberrant TDP-43 phase transition in the cytosol caused by ODN uptake could impair nucleocytoplasmic trafficking, further enhancing TDP-43 pathology.

Short TDP-43 isoforms or fragments, generated through the translation of alternatively spliced isoforms or proteolytic cleavage, are involved in TDP-43 pathology (79). We have found that the levels of a 35-kDa species of TDP-43, denoted as TDP-35 here, were increased upon ODN treatment (Figs. 7*C*, 8, *E* and *F*). Among the reported TDP-43 short isoforms, an N-terminally truncated form with a molecular weight of 35-kDa, termed Met85-TDP-35 previously (39), is generated through 91 bp skipping in *Tardbp* exon 2 using the non-canonical splicing pair (UU: AG) and alternative translational initiation at methionine-85 (39). Herein, we found that this 91 bp deleted *Tardbp* isoform is expressed in the RAW264.7 cells and primary microglia (Fig. S3). However, it remains to be determined whether the 35-kDa TDP-43 species we have detected in these cells is the previously identified Met85-TDP-35 or the 35-kDa fragment of TDP-43 resulting from proteolytic cleavage. Given that short isoforms or fragments of TDP-43, including Met85-TDP-35, can sequester the full-length TDP-43 into the cytoplasm as shown previously (38, 39, 80) and TDP-35 can bind to the ODN (Fig. 7*D*), the accumulation of TDP-35 in the cytosol might cause more TDP-43 retention in the cytoplasm by interacting with DNA and full-length TDP-43. However, due to the lack of antibodies specifically recognizing TDP-35, we were not able to test this speculation. Nevertheless, our studies provide strong evidence that TDP-43 protein homeostasis is disrupted by the cytoplasmic accumulation of DNA, which may be one of the mechanisms leading to TDP-43 proteinopathy seen in neurodegenerative diseases.

## Experimental procedures

### Oligodeoxynucleotides (ODNs)

CpG-ODN 2216 (IAX-200–005-C100), 2006 (IAX-200–006-C100), and inhibitory ODN (iODN) (IAX-200–052-C100) were ordered from AdipoGen Life Science. Other oligodeoxynucleotides (ODNs) used in this study were synthesized by Integrated DNA Technologies (IDT). The sequences of the ODNs used in this study are shown in Table S1. All the single-stranded ODNs were modified with phosphodiester backbone. Single strains of ODNs were thermally annealed to form dsDNA. For the ODN treatment in all the cell lines used in this study, 1 μM of the ODNs were directly added to the cell culture medium unless indicated otherwise.

### Plasmids and small hairpin RNAs

The full-length, N-terminal (amino acid 1–92), RNA recognition motif (amino acids 93–266), and C-terminal domains (amino acids 267–414) of human TDP-43 were cloned into pEGFP-C1 vector with a C-terminal GFP tag.

Small hairpin RNAs (shRNAs) targeting the mouse *Tardbp* gene and the nontargeting shRNA oligos synthesized by Thermo Fisher Scientific were cloned into PLKO.1 lentiviral vector. The targeted sequences of shTDP-43-1 and −2 were 5′-GGTATATGTTGTCAACTATCC-3′ and 5′-GTAGATGTCTTCATTCCCAAA-3′, respectively. Viral packaging plasmids psPAX2 and pMD2.G together with lentiviral shRNA plasmids were transfected to HEK293T cells in a 2:1:3 ratio using polyethylenimine (PEI) following the manufacturer’s guideline. Lentiviruses were collected 72 h after transfection and filtered with 0.45 μm filters. For transduction in RAW264.7 cells, the cells were infected with the lentivirus for 48 to 72 h and the knockdown efficiency of TDP-43 was verified by Western blot.

### Primary antibodies and reagents

The following antibodies were used in this study: Mouse anti-TDP43 (R&D systems, MAB7778, epitope amino acids 1–103, 1:5000 for Western blot and 1:500 for immunostaining), Rabbit anti-TDP43 (Proteintech Group, 12892-1-AP, epitope amino acids 288–414, 1:5000 for Western blot), mouse anti-GAPDH (Proteintech Group, 60004-1-Ig, 1:5000 for Western blot), Rabbit anti-G3BP1 (Proteintech Group, 13057-2-AP, 1:300 for immunostaining), Rabbit anti-FUS (Proteintech Group, 11570-1-AP, 1:300 for immunostaining), Rabbit anti-GFAP (Proteintech Group, 16825-1-AP, 1:300 for immunostaining), rabbit anti-MAP2 (Proteintech Group, 17490-1-AP, 1:300 for immunostaining), rabbit anti-IBA-1 (Wako, 01,919,741, 1:300 for immunostaining), rat anti-mouse LAMP1 (1D4B) (BD Biosciences, 553,793, 1:300 for immunostaining), rabbit anti-EEA1 (Cell signaling, #3288, 1:200 for immunostaining), rabbit anti-phspho-Histone (S139) H2AX antibody (R&D systems, 4418-APC-100, 1:300 for immunostaining), rabbit anti-phospho-TDP-43 (Ser409/410) (Proteintech group, 80007-1-RR, 1:5000 for Western blot), rabbit anti-LaminB1 (Proteintech group, 66095-1-Ig, 1:5000 for Western blot), rabbit anti-KPNB1 (Proteintech group, 10077-1-AP, 1:3000 for Western blot and 1:300 for immunostaining), and rabbit anti-CRM1 (Proteintech group, 27917-1-AP, 1:5000 for Western blot and 1:300 for immunostaining).

The following reagents were also used in the study: Dulbecco’s modified Eagle’s medium (DMEM) (Cellgro, 10–017-CV), HBSS (21–020-CV; Cellgro), DMEM/Ham’s F-12, (DMEM/F-12) (10–092-CV; Cellgro), 0.25% Trypsin (Corning, 25–053-CI), protease inhibitor (Roche, 05,056,489,001), Pierce BCA Protein Assay Kit (Thermo Scientific, 23,225), DNA damage inducer Etoposide (TCI America, E0675100 MG), PP2 (AdipoGen Life Sciences, AG-CR1-3563), Wortmannin (AdipoGen Life Sciences, AG-CN2-0023), Nu7441 (Apexbio Technology, 501,013,746), Lipopolysaccharides (LPS) (Sigma, L2654), polyinosinic-poly(C) potassium salt [poly(I: C)] (Sigma, 31,852–29–6), Imiquimod (Calbiochem, 99,011–02–6).

### Cell culture

Mouse macrophage cell line RAW264.7 and mouse motor neuron-like hybrid cell line NSC34 were also used in this study. A Toll-like receptor 9 (TLR9) deficient macrophage cell line was originally derived from transformed primary bone marrow-derived macrophages from TLR9 deficient mice (BEI Resources NR9569). The *Lmna*^−/−^ mouse embryonic fibroblasts (MEFs) were derived from *Lmna*^−/−^ mice, originally developed by the laboratory of Colin Stewart (81), and spontaneously immortalized through repeated passage (82). All the cells above were maintained in DMEM supplemented with 10% fetal bovine serum (Sigma-Aldrich) and 1% penicillin penicillin-streptomycin (Invitrogen) in a humidified incubator at 37 °C and 5% CO2. All the cells used in this study were subjected to *mycoplasma* tests and cells were free from *mycoplasma* contamination for all experiments.

TDP-43^Q331K^ mice (83) in C57BL/6 background were obtained from the Jackson Laboratory. WT and TDP-43 Q331K primary microglia were isolated from P0 to P2 pups. Brains were collected and digested in 0.05% trypsin for 10 min. The digested brains were plated in DMEM/F12 supplemented with 10% FBS, 1% penicillin-streptomycin, and 5 ng/ml of GM-CSF. After 2 weeks of culture, flasks were gently shaken for 1 h at 200 rpm, and the microglia containing supernatants were harvested and plated for the ODN treatment. WT primary cortical neurons and astrocytes were isolated from P0 to P2 pups and cultured according to the published protocols (84, 85). All animal experiments and procedures were performed according to NIH guidelines and were approved by the Institutional Animal Care and Use Committee at Cornell.

### Immunofluorescence staining, image acquisition, and analysis

Cells were fixed with 4% paraformaldehyde (PFA) for 10 min, treated with 0.1% (vol/vol) Triton X-100 for 10 min or 0.05% saponin for 30 min as indicated in the figure legends, and then incubated with Odyssey blocking buffer (LI-COR Biosciences, 927–40000) for 1 h at room temperature. Samples were then incubated with the indicated primary antibody overnight, followed by incubation with the appropriate Alexa Fluor-conjugated secondary antibody, and stained with Hoechst to visualize the nucleus. Images were acquired using a confocal microscope (Intelligent Imaging Innovations) or Leica microscope. Confocal images were taken under a 3D model and were processed in a maximum-intensity projection. For image quantification, 40x confocal images in a random field view from each coverslip were quantified. To quantify the colocalization of TDP-43 with the indicated proteins in the cytoplasm, each cell was individually selected using the ROI tool and analyzed using the Coloc2 plugin in ImageJ. Pearson’s correlation coefficient (r) value was used to determine the colocalization efficiency (86, 87). Pearson’s r values closer to one indicate a strong positive correlation (high colocalization), while values closer to 0 indicate no correlation (no colocalization).

### DNA pull-down assay

Biotinylated ODNs were synthesized by IDT. RAW264.7 cells were lysed in 50 mM Tris pH 8.0, 150 mM NaCl, 1% Triton, and 0.1% deoxycholic acid with protease inhibitors. The cell lysates were mixed with ODNs and incubated for 4 h at 4 °C. The streptavidin resin (Genscript, L00353) was washed three times with wash buffer (50 mM Tris pH 8.0, 150 mM NaCl, 1% Triton) and then were added to the lysates for 2 h of incubation at 4 °C. Beads were washed four times with wash buffer and then resuspended in 2 × SDS loading buffer, and the bound proteins were resolved by SDS-PAGE, followed by immunoblotting with the antibodies to TDP-43.

### Purification of recombinant TDP-43 protein

TDP-43 tagged with glutathione S-transferase (GST) at the N-terminus was expressed in BL21 E.coli at 18 °C for 16 h after induction by adding 0.5 mM IPTG. In brief, cells were harvested by centrifugation at 4000 rpm for 15 min at 4 °C. The cell pellet was resuspended in buffer A [50 mM Tris-HCL (pH 7.5), 150 mM NaCl, and 5 mM dithiothreitol (DTT) containing 0.1% Triton-X 100 and protease inhibitor cocktail], sonicated, and ultracentrifuged at 40,000 rpm for 1 h at 4 °C. The soluble fractions were transferred to the columns with GST fast-flow beads (GE Healthcare, 17–5132–02). The columns were washed with buffer A and eluted with buffer B [200 mM Tris-HCL (pH 7.5), 150 mM NaCl, 1 mM dithiothreitol (DTT), and 50 mM glutathione]. The purified proteins were confirmed by Coomassie brilliant blue staining and Western blot before use.

### *In vitro* phase separation assay

For the *in vitro* phase separation assay, purified TDP-43 protein and ODNs were diluted in LLPS buffer (50 mM HEPES, 150 mM NaCl, 5 mM DTT, 5% glycerol, pH 7.5) to a molar ratio 1:0.5 (TDP-43: ODNs). The above mixture was then added to LLPS buffer and incubated in a 96-well plate for 30 min at room temperature. The formation of liquid droplets was examined by using a Leica microscope with differential interference contrast (DIC). To quantify the number and size of liquid droplets, the liquid droplets were manually selected and quantified using Image J.

### Urea insoluble fraction and Western blot analysis

Cells were lysed by RIPA buffer (150 mM NaCl, 50 mM Tris-HCl, 1% Triton X-100, 0.5% sodium deoxycholate, 0.1% SDS, pH 8.0) with protease inhibitor. After centrifugation at 14,000*g* for 10 min at 4 °C, supernatants were collected as the RIPA-soluble fraction. The insoluble pellets were washed with RIPA buffer and extracted in 2 × v/w of Urea buffer (7 M Urea, 2 M Thiourea, 4% CHAPS, 30 mM Tris, pH 8.5). After sonication, samples were centrifuged at 200,000*g* at 24 °C for 1 h and the supernatant was collected as the Urea-soluble fraction. The soluble and insoluble fractions were subsequently analyzed by Western blot.

### Nuclear and cytoplasmic fractionation

RAW264.7 cells or NSC34 cells in a 6-well plate treated accordingly were harvested and lysed with 200 μl of cytoplasmic extraction buffer [10 mM HEPES, 10 mM KCl, 2 mM Mg(Ac)2, 3 mM CaCl2, 340 mM sucrose, 1 mM dithiothreitol (DTT), pH 7.9] on ice for 20 min, followed by the addition of NP-40 (Amresco) to a final concentration of 0.1% (vol/vol). Samples were then vortexed for 15 s and centrifuged for 10 min at 3500*g* at 4 °C. Supernatants (the cytoplasmic fraction) were collected and stored at −80 °C. Pellets were dissolved in 50 μl of RIPA buffer, incubated on ice for 10 min, and then centrifuged at 14,000*g* for 10 min at 4 °C. Supernatants (the nuclear fraction) were collected and stored at −80 °C.

### PCR with reverse transcription (RT-PCR)

Total RNA was extracted from RAW264.7 cells or primary microglia using TRIzol (Thermo Scientific). One microgram of total RNA was reverse transcribed to cDNA using SuperScript III Reverse Transcriptase (Invitrogen) according to the manufacturer’s protocol. PCR was conducted with 1 μl cDNA input in a 20 μl reaction using Taq polymerase, with the following cycling parameters: initial denaturation: 94 °C for 5 min; 35 cycles: 94 °C for 15 s, 58 °C for 15 s, 72 °C for 15 s; final extension: 72 °C for 5 min. The resulting products were visualized on a 1.5% TAE gel. The primers used to identify the TDP-43 splice variant TDP-35 were F1 (5′-ATTCGGGTAACAGAAGATG-3′) and R1 (5′-GAGACCCAACACTATGAGG-3′).

### Statistical analysis

All statistical analyses were performed using GraphPad Prism 9 or 10. All data are presented as mean ± SEM. Statistical significance was assessed by unpaired two-tailed student’s *t* test. P values less than or equal to 0.05 were considered statistically significant. ∗*p* < 0.05; ∗∗*p* < 0.01; ∗∗∗*p* < 0.01.

## Data availability

The data supporting the findings of this study are included in the figures or supplemental material. Additional data is available from the corresponding author on request. No data is deposited in databases.

## Supporting information

This article contains supporting information (20, 21, 24, 25, 88, 89).

## Conflict of interests

The authors declare that they have no conflicts of interest with the contents of this article.
