# Supplementary material for: Regulation of TAR DNA binding protein 43 (TDP-43) homeostasis by cytosolic DNA accumulation
Source: J Biol Chem. 2024 Nov 15;300(12):107999. doi: 10.1016/j.jbc.2024.107999 (PMC11719319; doi:10.1016/j.jbc.2024.107999)
Supplement: Supplementary file 1 [file mmc1.pdf]

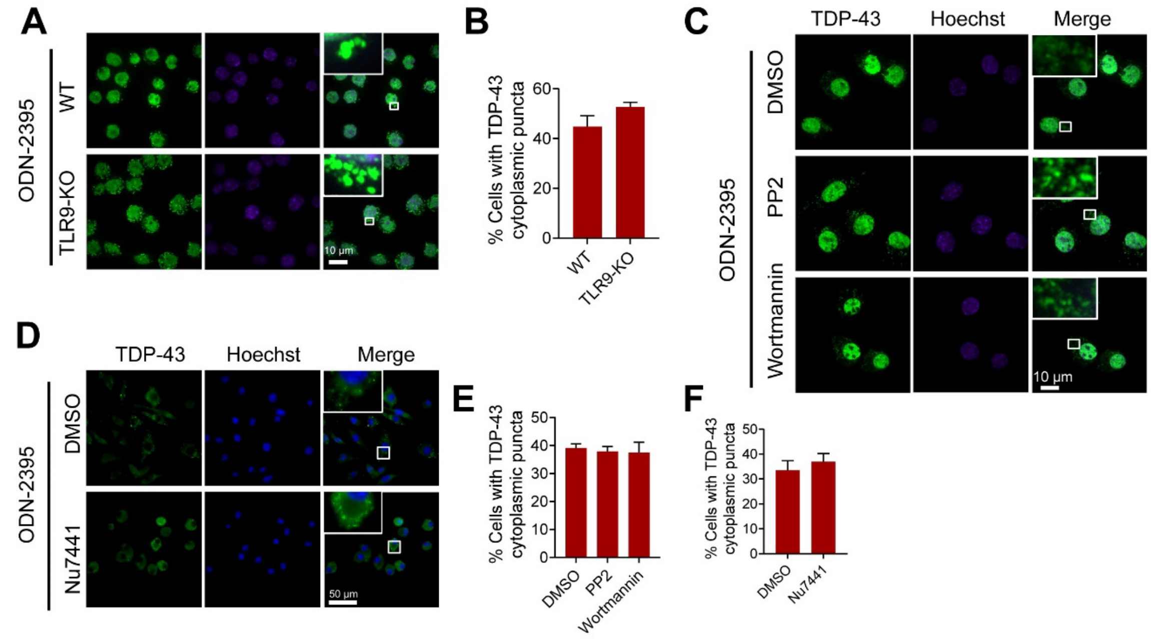

Fig.S1: CpG-ODN-induced TDP-43 cytoplasmic puncta formation is independent of CpG-ODN mediated signaling pathways, related to Figure 1. (A, B) CpG-ODN treatment induces TDP-43 cytoplasmic puncta formation in the TLR9 deficient cells. Wild-type (WT) and TLR9 knockout (TLR9-KO) macrophages were treated with CpG-ODN 2395 for 24 h. Cells were fixed, permeabilized with Triton-X100, and stained with anti-TDP-43 antibodies. Representative images were shown for each condition. Scale bar, 10 µm. The percentage of the cells with cytoplasmic TDP-43 puncta was quantified (B). Data are presented as means of ± SEM from 3 independent experiments (n=3). **(C-F)** SFK inhibitor PP2, PI3K inhibitor wortmannin, and DNA-PK inhibitor Nu7441 do not affect the formation of TDP-43 cytoplasmic puncta triggered by CpG-ODN. RAW264.7 cells were pretreated with 1.0 µM of PP2, wortmannin, or Nu7441 for 2 h and the cells were then treated with CpG-ODN 2395 for 24 h. Cells were fixed, permeabilized with Triton-X100 (C) or saponin (D), and stained with anti-TDP-43 antibodies. Representative images were shown for each condition. Scale bar, 10 µm (C); 50 µm (D). The percentage of the cells with cytoplasmic TDP-43 puncta was quantified (F, F). Data are presented as means of ± SEM from 3 independent experiments (n=3).

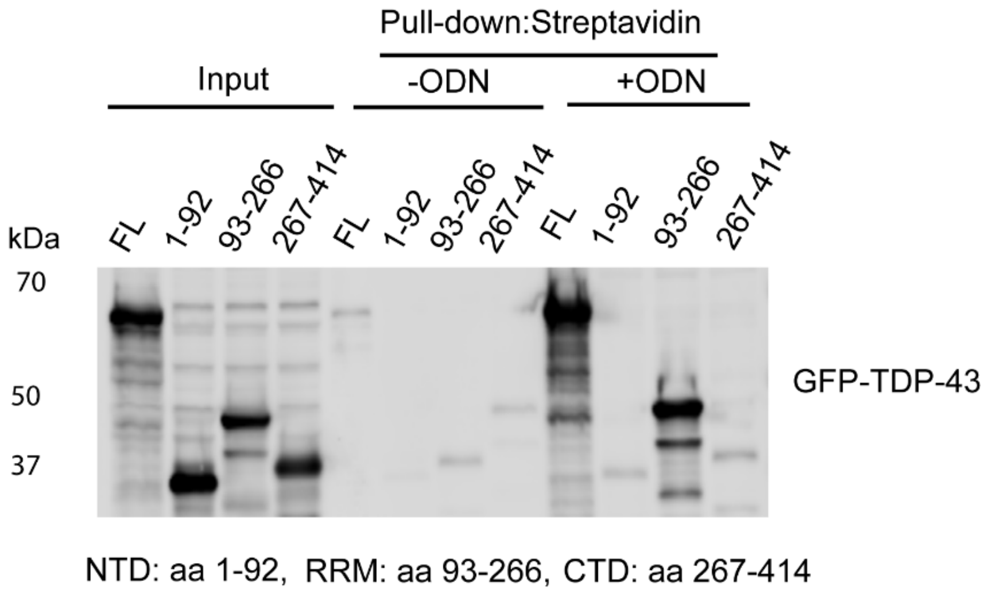

Fig.S2: RNA binding domains of TDP-43 mediate TDP-43 binding to DNA. HEK293T cells were transfected with the GFP-targeted full-length TDP-43 (FL) or truncated TDP-43 mutants, and cell lysates were incubated with biotinylated ODNs followed by western blot to detect the TDP-43-ODN binding. N-terminal (NTD): amino acids 1-92, RNA recognition motif (RRM): amino acids 93-266, and C-terminal (CTD): amino acids 267-414.

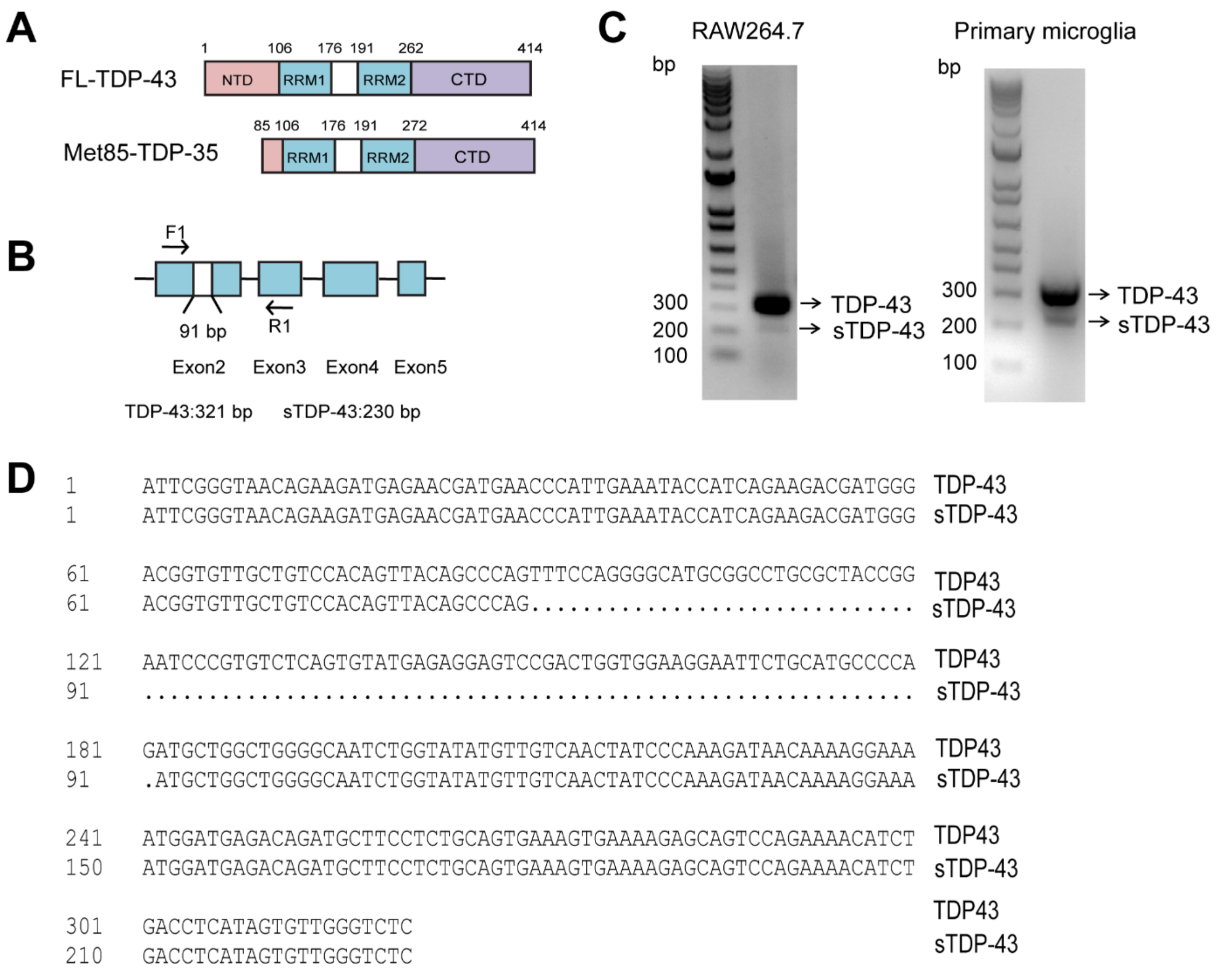

Fig.S3: The 91 bp deleted *Tardbp* isoform is expressed in the RAW264.7 cells and primary microglia. **(A)** Domain structure of full-length TDP-43 and Met85-TDP-35 identified by previous study. NTD: N-terminal domain, RRM: RNA recognition motif, CTD: C-terminal domain. **(B)** The structure of *Tardbp* gene exon 2-5. Blue boxes indicate exons. The white box in exon 2 denotes the 91 bp skipped by alternative splicing. **(C)** RT-PCR amplification of the TDP-43 splice variant (sTDP-43) using RNA isolated from RAW264.7 cells and primary microglia. Primer pair F1 and R1 targeting exons 2 and 3 of the *Tardbp* gene, respectively, yields PCR fragments of 321 (transcript for full-length TDP-43) or 230 bp (91 bp deleted transcript). **(D)** Sequence alignment of the transcripts of TDP-43 and the TDP-43 splice variant. The PCR fragments were sequenced by using the primer pair F1 and R1. The 91 bp splicing deletion is signified by dotted lines. Upper line: full-length TDP-43, lower line: sTDP-43.

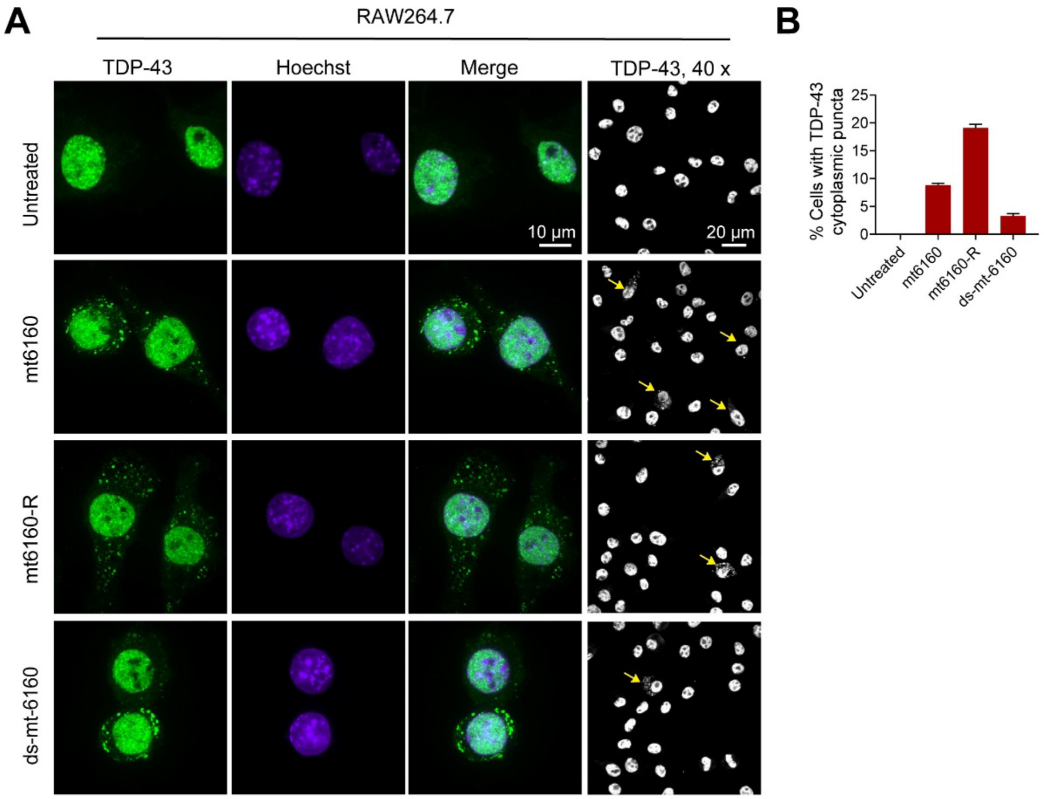

Fig.S4: Short mtDNA induces the TDP-43 cytoplasmic puncta formation. (A, B) RAW264.7 cells were untreated or treated with the ODN containing mitochondrial DNA sequence (mt6160), the reverse-complementary ODN mt6160-R, or double-stranded mt6160 for 24 h, fixed, permeabilized with Triton-X100, and stained with mouse anti-TDP-43 antibodies. Representative images from three independent experiments were shown for each condition. Scale bar, 10 μm. The percentage of the cells with cytoplasmic TDP-43 puncta was quantified (B). Data are presented as means of ± SEM from 3 independent experiments (n=3).

Table.S1 The ODNs used in this study

ODNs	Sequence (5' to 3')	Description	Length (mer/bp)	TDP-43 cytoplasmic puncta formation	TDP-43 binding	Cellular uptake
2395	tcgtcgttttcggcgcgcgccg	Type C CpG-ODN[1]	22	Yes	Yes	Yes
2395-R	cggcgcgcgccgaaaacgacga	Reverse-complementary ODN of 2395	22	Yes		
2395-ctl	tgctgcttttgggggggcccccc	Control ODN for CpG-ODN 2395[2]	22	Yes	Yes	Yes
2395-ctl-R	ggggggccccccaaaagcagca	Reverse-complementary ODN of 2395-ctl	22	Yes		
mt6160	cccccgatatggcgtttcccc	Mitochondrial DNA segment harboring a CpG motif[3]	21	Yes		
mt6160-R	ggggaaacgccatatcggggg	Reverse-complementary ODN of mt6160	21	Yes		
(CA)11	cacacacacacacacacacaca	CA-repeated ODNs that bind to TDP-43 with very low affinity	22	No	Yes, very low affinity	Yes, very low efficiency
(TG)11	tgtgtgtgtgtgtgtgtgtgtg	TG-repeated ODNs that have high binding affinity to TDP-43[4-6]	22	Yes	Yes	Yes, very low efficiency
ds-2395-ctl			22	Yes	Yes	Yes
ds-2395			22	Yes	Yes	Yes
ds-mt6160			21	Yes		

References

1. Sivori, S., Carlomagno, S., Moretta, L., and Moretta, A. (2006). Comparison of different CpG oligodeoxynucleotide classes for their capability to stimulate human NK cells. Eur J Immunol *36*, 961-967. 10.1002/eji.200535781.
2. Bauer, S., Kirschning, C.J., Hacker, H., Redecke, V., Hausmann, S., Akira, S., Wagner, H., and Lipford, G.B. (2001). Human TLR9 confers responsiveness to bacterial DNA via species-specific CpG motif recognition. Proc Natl Acad Sci U S A *98*, 9237-9242. 10.1073/pnas.161293498.
3. Ries, M., Schuster, P., Thomann, S., Donhauser, N., Vollmer, J., and Schmidt, B. (2013). Identification of novel oligonucleotides from mitochondrial DNA that spontaneously induce plasmacytoid dendritic cell activation. J Leukoc Biol *94*, 123-135. 10.1189/jlb.0612278.
4. Kuo, P.H., Chiang, C.H., Wang, Y.T., Doudeva, L.G., and Yuan, H.S. (2014). The crystal structure of TDP-43 RRM1-DNA complex reveals the specific recognition for UG- and TG-rich nucleic acids. Nucleic Acids Res *42*, 4712-4722. 10.1093/nar/gkt1407.
5. Furukawa, Y., Suzuki, Y., Fukuoka, M., Nagasawa, K., Nakagome, K., Shimizu, H., Mukaiyama, A., and Akiyama, S. (2016). A molecular mechanism realizing sequence-specific recognition of nucleic acids by TDP-43. Sci Rep *6*, 20576. 10.1038/srep20576.
6. Kitamura, A., Shibasaki, A., Takeda, K., Suno, R., and Kinjo, M. (2018). Analysis of the substrate recognition state of TDP-43 to single-stranded DNA using fluorescence correlation spectroscopy. Biochem Biophys Rep *14*, 58-63. 10.1016/j.bbrep.2018.03.009.
